# Identification of key genes and pathways affected in epicardial adipose tissue from patients with coronary artery disease by integrated bioinformatics analysis

**DOI:** 10.7717/peerj.8763

**Published:** 2020-03-25

**Authors:** Liao Tan, Qian Xu, Qianchen Wang, Ruizheng Shi, Guogang Zhang

**Affiliations:** 1 Department of Cardiovascular Medicine, Xiangya Hospital, Central South University, Changsha, China; 2 Institute of Hypertension, Central South University, Changsha, China; 3 Department of Cardiovascular Surgery, Xiangya Hospital, Central South University, Changsha, China; 4 Department of Cardiovascular Medicine, The Third Xiangya Hospital, Central South University, Changsha, China

**Keywords:** Bioinformatics analysis, Epicardial adipose tissue, Gene expression omnibus, Coronary artery disease, Subcutaneous adipose tissue

## Abstract

**Background:**

Coronary artery disease (CAD) is a common disease with high cost and mortality. Here, we studied the differentially expressed genes (DEGs) between epicardial adipose tissue (EAT) and subcutaneous adipose tissue (SAT) from patients with CAD to explore the possible pathways and mechanisms through which EAT participates in the CAD pathological process.

**Methods:**

Microarray data for EAT and SAT were obtained from the Gene Expression Omnibus database, including three separate expression datasets: GSE24425, GSE64554 and GSE120774. The DEGs between EAT samples and SAT control samples were screened out using the limma package in the R language. Next, we conducted bioinformatic analysis of gene ontology terms and Kyoto Encyclopedia of Genes and Genomes pathways to discover the enriched gene sets and pathways associated with DEGs. Simultaneously, gene set enrichment analysis was carried out to discover enriched gene functions and pathways from all expression data rather than DEGs. The PPI network was constructed to reveal the possible protein interactions consistent with CAD. Mcode and Cytohubba in Cytoscape revealed the possible key CAD genes. In the next step, the corresponding predicted microRNAs (miRNAs) were analysed using miRNA Data Integration Portal. RT-PCR was used to validate the bioinformatic results.

**Results:**

The three datasets had a total of 89 DEGs (FC log2 > 1 and *P* value < 0.05). By comparing EAT and SAT, ten common key genes (HOXA5, HOXB5, HOXC6, HOXC8, HOXB7, COL1A1, CCND1, CCL2, HP and TWIST1) were identified. In enrichment analysis, pro-inflammatory and immunological genes and pathways were up-regulated. This could help elucidate the molecular expression mechanism underlying the involvement of EAT in CAD development. Several miRNAs were predicted to regulate these DEGs. In particular, hsa-miR-196a-5p and hsa-miR-196b-5p may be more reliably associated with CAD. Finally, RT-PCR validated the significant difference of OXA5, HOXC6, HOXC8, HOXB7, COL1A1, CCL2 between EAT and SAT (*P* value < 0.05).

**Conclusions:**

Between EAT and SAT in CAD patients, a total of 89 DEGs, and 10 key genes, including HOXA5, HOXB5, HOXC6, HOXC8, HOXB7, COL1A1, CCND1, CCL2, HP and TWIST1, and miRNAs hsa-miR-196a-5p and hsa-miR-196b-5p were predicted to play essential roles in CAD pathogenesis. Pro-inflammatory and immunological pathways could act as key EAT regulators by participating in the CAD pathological process.

## Introduction

Atherosclerosis is a chronic artery disease that is the major cause of coronary artery disease (CAD) and stroke. It is a leading cause of death worldwide (Herrington et al., 2016; Jia et al., 2014; Zhang, Lai & Jia, 2015). In recent years, visceral adipose tissue (VAT) has been identified as affecting atherosclerosis and CAD pathogenesis persistently releasing pro-inflammatory and decrease anti-inflammatory adipokines into the circulation via a paracrine or endocrine pathway (Alexopoulos, Katritsis & Raggi, 2014; Wu & Zhao, 2006). Epicardial adipose tissue (EAT) is one of the most important VAT components. EAT, due to its position close to the myocardium, which shares the same microcirculation as the coronary artery, has a unique effect on CAD (Nakanishi et al., 2014; Patel et al., 2016).

So far, clinical studies have shown that EAT expansion is an independent CAD risk factor (Herrington et al., 2016; Mahabadi et al., 2013; Nakanishi et al., 2014; Liu et al., 2019). Some EAT functions were reported in cell and animal studies. EAT releases some factors (apelin (Yao et al., 2015), adiponectin (Ouchi & Walsh, 2008), MCP-1 (Niu & Kolattukudy, 2009)) that effect the myocardium and coronary arteries via paracrine and vasocrine pathways. Furthermore, exosomes released from EAT could carry molecules (proteins, RNA and lipids) that establish cross-talk between the pathological EAT and the coronary artery (Thomou et al., 2017). The NALP3/inflammasome pathway is activated by microbial colonisation in EAT in CAD patients (Guauque-Olarte et al., 2011). The expression of proteins involved in oxidative stress, metabolism regulation, gene transcription regulation, and angiogenesis were significantly increased in CAD patient EAT (Vacca et al., 2016).

However, it is not completely understood how EAT participates in CAD. One of the major limitations in studying EAT function is that only patients undergoing cardiac surgery are studied. Collecting EAT from healthy subjects is not possible for obvious ethical reasons. Therefore, subcutaneous adipose tissue (SAT) was always used as the second-best control in previous studies (Gruzdeva et al., 2017; Salgado-Somoza et al., 2010; Hirata et al., 2011).

We attempted to verify the pre-existing function and predict a new effect of EAT in pathological CAD processes. Here, microarray analysis of EAT and SAT was conducted based on three independent expression array databases. These gene expression features could help discover new CAD biomarkers and pioneer therapeutic strategies.

## Materials and Methods

### Data source

The microarray expression data sets (GSE24425, GSE64554 and GSE120774) were downloaded from the Gene Expression Omnibus (GEO) database. A total of 28 EAT samples and 27 SAT control samples were collected from the same CAD patients undergoing coronary artery bypass grafting surgeries (CABG). GSE24425 used the Illumina HumanWG-6 V3.0 expression beadchip containing six paired EAT and SAT samples. GSE64554 used the Illumina HumanHT-12 V3.0 expression beadchip. GSE120774 used the HuGene-1_0-st Affymetrix Human Gene 1.0 ST Array, including 13 EAT samples with EAT controls and nine EAT samples with eight SAT controls, respectively. The three datasets were all quantile normalised and log2-transformed.

### Data pre-processing

Data pre-processing consisted of three units: transition from gene probes to gene symbols, data consolidation, and batch normalisation. First, the three series matrix files were annotated with an official gene symbol using the data table of the microarray platform, and the gene expression matrix files were obtained. The three gene expression matrix files were merged into one file using a Perl script. Gene probes without gene symbols or genes with more than one probe were eliminated or averaged, respectively. To ensure the integrity and comparability of the datasets, the batch normalisation of merged data was pre-processed by sva package (Leek et al., 2012) using the R language (R Development Core Team, 2018). Batch effects are the most widely recognised potential latent variable in genomic experiments. The sva package was used to eliminate the latent variables or unwanted heterogeneity in the high-throughput data. The batch normalisation including two steps: identification of potential impact factors and elimination of batch effects using the ComBat function.

### Differentially expressed gene analysis

The differentially expressed genes (DEGs) between EAT samples and SAT samples were determined using the limma package in R. The thresholds were log2 (fold change) > 1 and *P* value < 0.05. DEG visualisation was done using a volcano map and heatmap using gglot2 (Ginestet, 2011) and the pheatmap package (Kolde, 2015).

### Functional enrichment analysis of DEGs

The Cluster Profiler R package (Yu et al., 2012) and the database for annotation, visualisation, and integrated discovery (DAVID 6.8, http://david.ncifcrf.gov) (Huang et al., 2007) were used to functionally analyse and analyse the enriched pathways of the key DEGs in gene ontology (GO) terms and Kyoto Encyclopedia of Genes and Genomes (KEGG) pathways. The *P* value was corrected using the Benjamini method or false discovery rate (FDR) for multiple testing calibrations. The threshold was *P* < 0.05.

### Gene set enrichment analysis

Gene set enrichment analysis (GSEA) (Subramanian et al., 2005) was performed using GSEA software. Gene sets used here were downloaded from the Molecular Signatures Database. Enrichment results satisfying a nominal *P*-value cut-off of < 0.05 with a FDR > 0.25 were considered statistically significant. MSigDB were download from http://software.broadinstitute.org/gsea/index.jsp (Liberzon et al., 2011).

### PPI network construction and analysis

To explore the interacting genes, the search tool (STRING 10.5; http://string-db.org) (Szklarczyk et al., 2015) was employed to establish a DEG PPI network, which was drawn using Cytoscape (Kohl, Wiese & Warscheid, 2011). Interaction with a combined score > 0.4 was set as the cut-off point. The most important module in the PPI network was identified using the plug-in Molecular Complex Detection (MCODE) (Bader & Hogue, 2003) of Cytoscape, an application to cluster a given network by topology, to find densely connected regions. The criteria for selection were as follows: MCODE scores > 5, degree cut-off = 2, node score cut-off = 0.2, max depth = 100, and *k*-score = 2. Subsequently, the maximal clique centrality (MCC) algorithm of CytoHubba (Chin et al., 2014) was used to explore the PPI network hub genes.

### Regulating miRNA prediction

The online prediction tool microRNA Data Integration Portal (mirDIP) (http://ophid.utoronto.ca/mirDIP) (Tokar et al., 2018) was used to predict potential microRNA (miRNA) targeting. Ten top hub genes were submitted, and the top five predicted miRNAs of every gene were chosen and listed.

### Assessment of the mRNA expression of hub genes using qRT-PCR

Eight EAT tissues and SAT tissues were collected from Xiangya Hospital. This study was approved by the Ethical Committee of Xiangya Hospital and conducted in accordance with the Declaration of Helsinki. In addition, each patient volunteered written informed consent. All tissues were immediately frozen in liquid nitrogen after resection and stored in liquid nitrogen. The clinical characteristics of included CAD patients were listed in Table 1.

**Table 1:** Clinical features of included CAD patients.

Clinical features of included patients	*n* = 8
Age	66.36 ± 8.33
Sex	
Male	6
Female	2
Heart function grade of NYHA	
I–II	6
III	2
IV	0
BMI (kg/m^2^)	23.01 ± 3.75
Hypertention	5
Diabetes	0
Hyperlipidemia	3
Smoking	4
Triple-vessel disease	8

Total RNA was extracted from tissues using TRIzol (Takara, Japan) according to the manufacturer’s instructions. Total RNA was reverse transcribed into cDNA using a cDNA Synthesis Kit (Takara, China). mRNA levels were tested using Nanodrop one (Thermo Fisher Scientific, Waltham, MA, USA). All reactions were performed on the Eppendorf Mastercycler ep realplex (2S; Eppendorf, Hamburg, Germany) using following cycling parameters: 95 °C for 30 s, followed by 40 cycles of 95 °C for 15 s, 60 °C for 34 s. RT-qPCR was performed using the FastStart Universal SYBR^®^ Green Master (ROX) (Takara, China). The primer sequences were listed in Data S1.

The relative expression level for each target gene was normalised by the Ct value of β-actin (internal control) using a 2^−ΔΔCt^ relative quantification method. A meaningful analysis between the two groups was performed by a paired *t*-test, and a *P* value < 0.05 was considered statistically significant.

## Results

### DEG analysis

**Figure 1:**
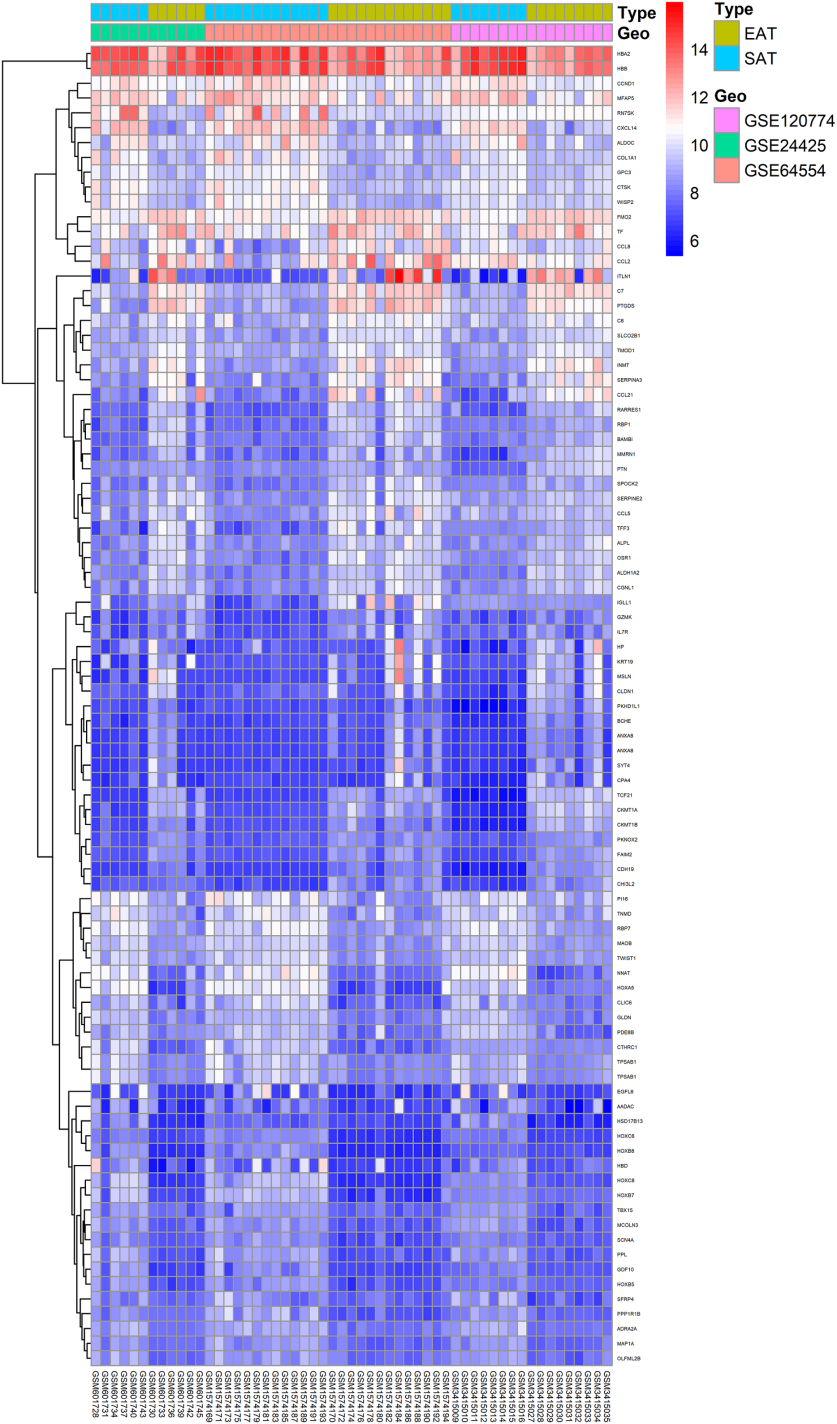
Heatmap of all DEGs between EAT and SAT. Each column represents a adipose tissue sample, and each row represents a DEG. The gradual colour change from blue to red indicates the changing process from downregulation to upregulation. DEGs, differentially expressed genes; EAT, epicardial adipose tissue; SAT, subcutaneous adipose tissue.

**Figure 2:**
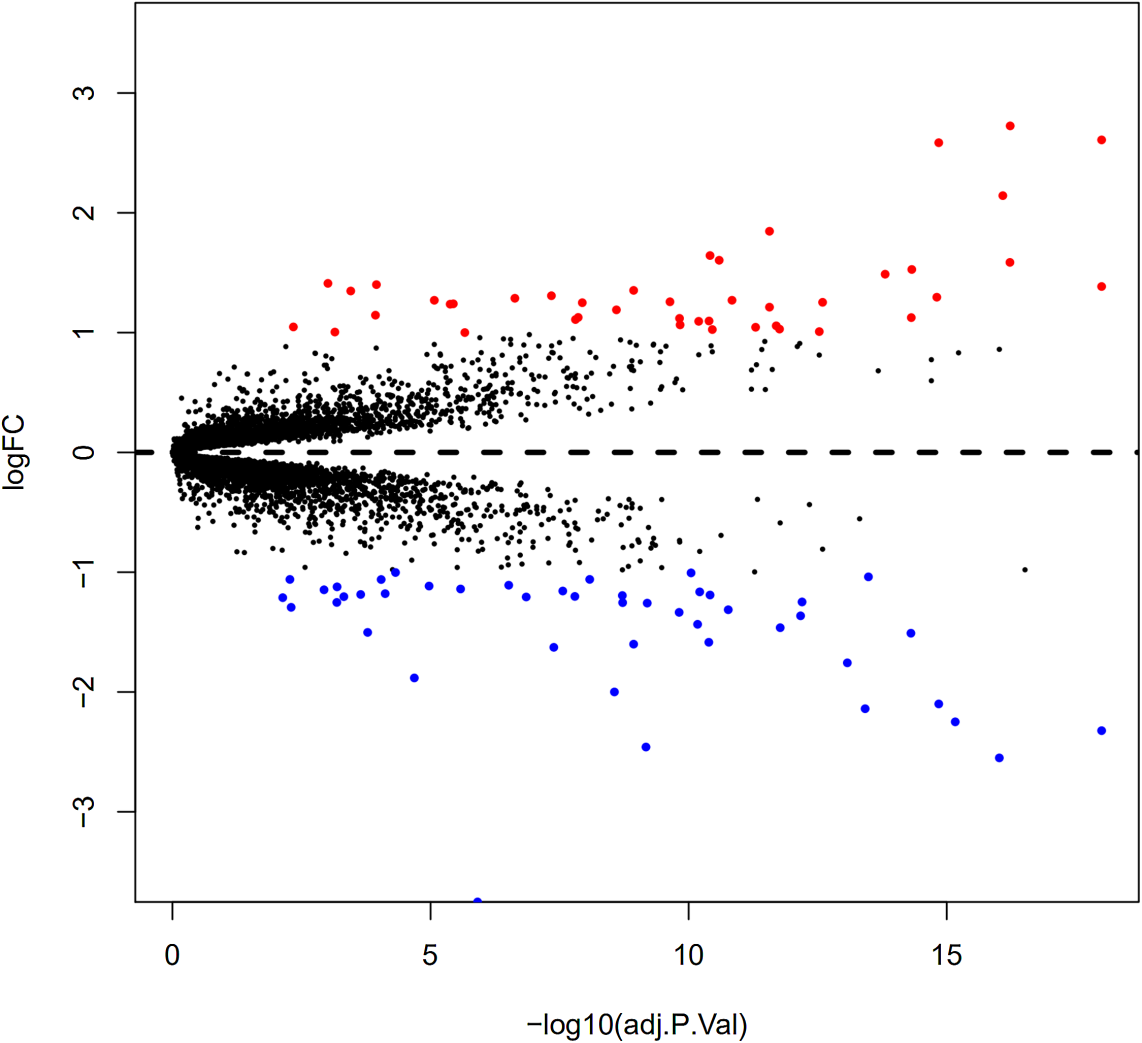
Volcano plot of 89 DEGs. Blue dot represent DEGs with fold change <1; red dot represent DEGs with fold change >1. *P* value < 0.05.

Here, 28 EAT samples and 27 SAT samples of CAD patients from the GSE24425, GSE64554 and GSE120774 datasets were analysed. After pre-processing, the raw data were merged and normalised (Fig. S1). Based on the cut-off criteria (adjusted *P* value < 0.05 and |log2 foldchange (FC)| > 1), a total of 89 DEGs were identified, including 43 up-regulated and 46 down-regulated DEGs. A DEG expression heat map and volcano map were shown in Figs. 1 and 2.

### Gene ontology enrichment analysis

**Figure 3:**
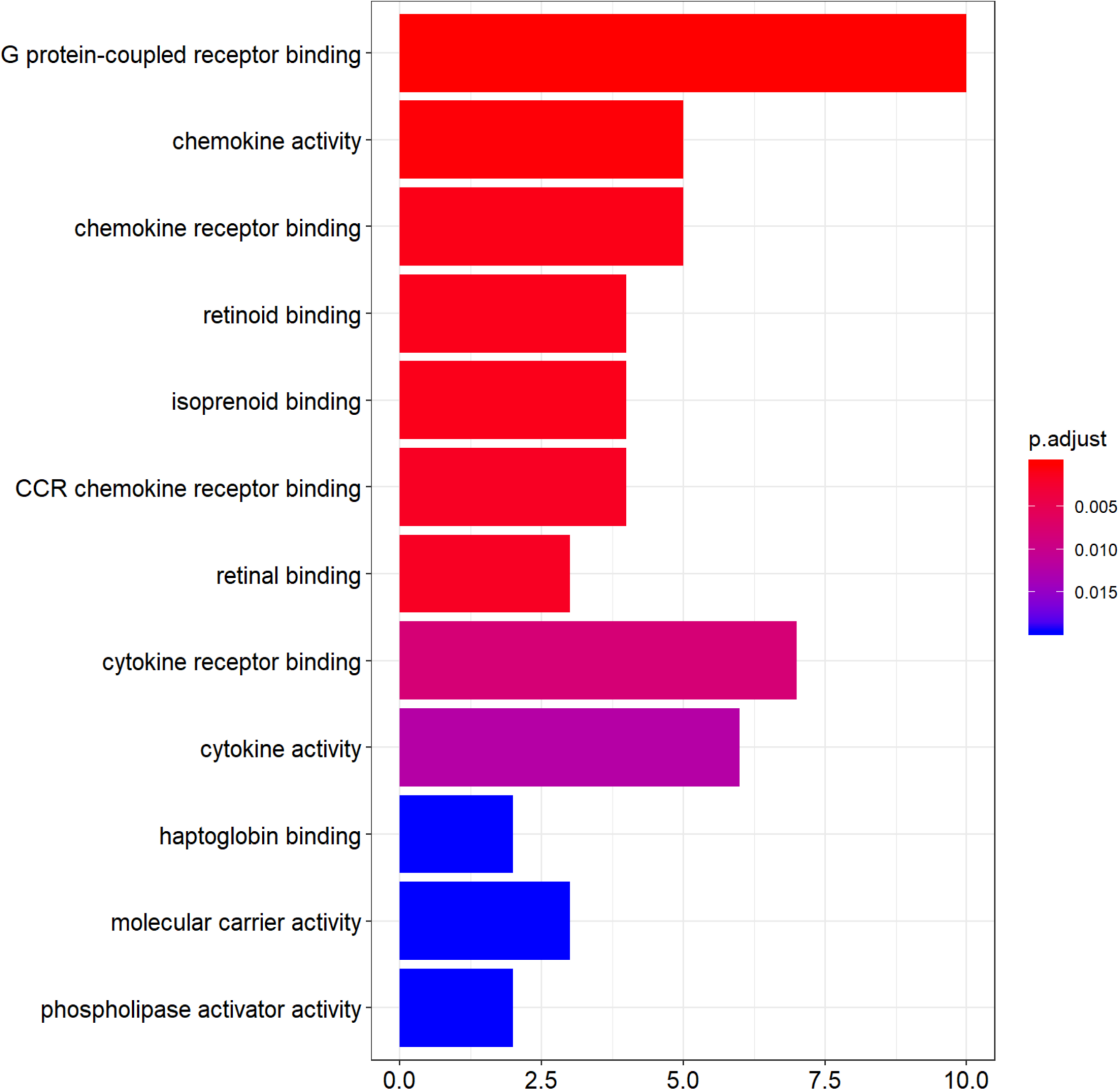
The top 12 significant GO terms enriched by DEGs. The *y*-axis labels represent clustered GO terms; *x*-axis represent the number of gene enriched in GO clusters. GO, gene ontology.

Functional enrichment analysis of DEGs was performed by using clusterProfiler package in the R language. The top 12 GO analysis results (Fig. 3) showed that genes were mainly enriched in G protein-coupled receptor binding, chemokine activity, chemokine receptor binding, retinoid binding, isoprenoid binding, CCR chemokine receptor binding, retinal binding, cytokine binding, cytokine activity, haptoglobin binding, molecular carrier activity, and phospholipase activator activity. All results were listed in Table S1.

### Pathway enrichment analysis

Significantly enriched pathways of DEGs are shown in Table 2 based on the DAVID online tool that was used to perform KEGG pathway analysis. Genes were mainly enriched in cytokine–cytokine receptor interaction, chemokine signalling pathway, prion diseases, malaria, and arginine and proline metabolism. Enriched pathways identified from DEGs were fewer but more reliable due to relatively limited DEGs from the three data sources.

**Table 2:** The significant KEGG pathways enriched by DEGs. *P* value < 0.05. KEGG: Kyoto Encyclopedia of Genes and Genomes.

Category term	Count	*P* value
hsa04060: Cytokine–cytokine receptor interaction	6	0.003647442
hsa04062: Chemokine signalling pathway	5	0.00818195
hsa05020: Prion diseases	3	0.009459285
hsa05144: Malaria	3	0.019044109
hsa00330: Arginine and proline metabolism	3	0.019784372

### Gene set enrichment analysis

**Figure 4:**
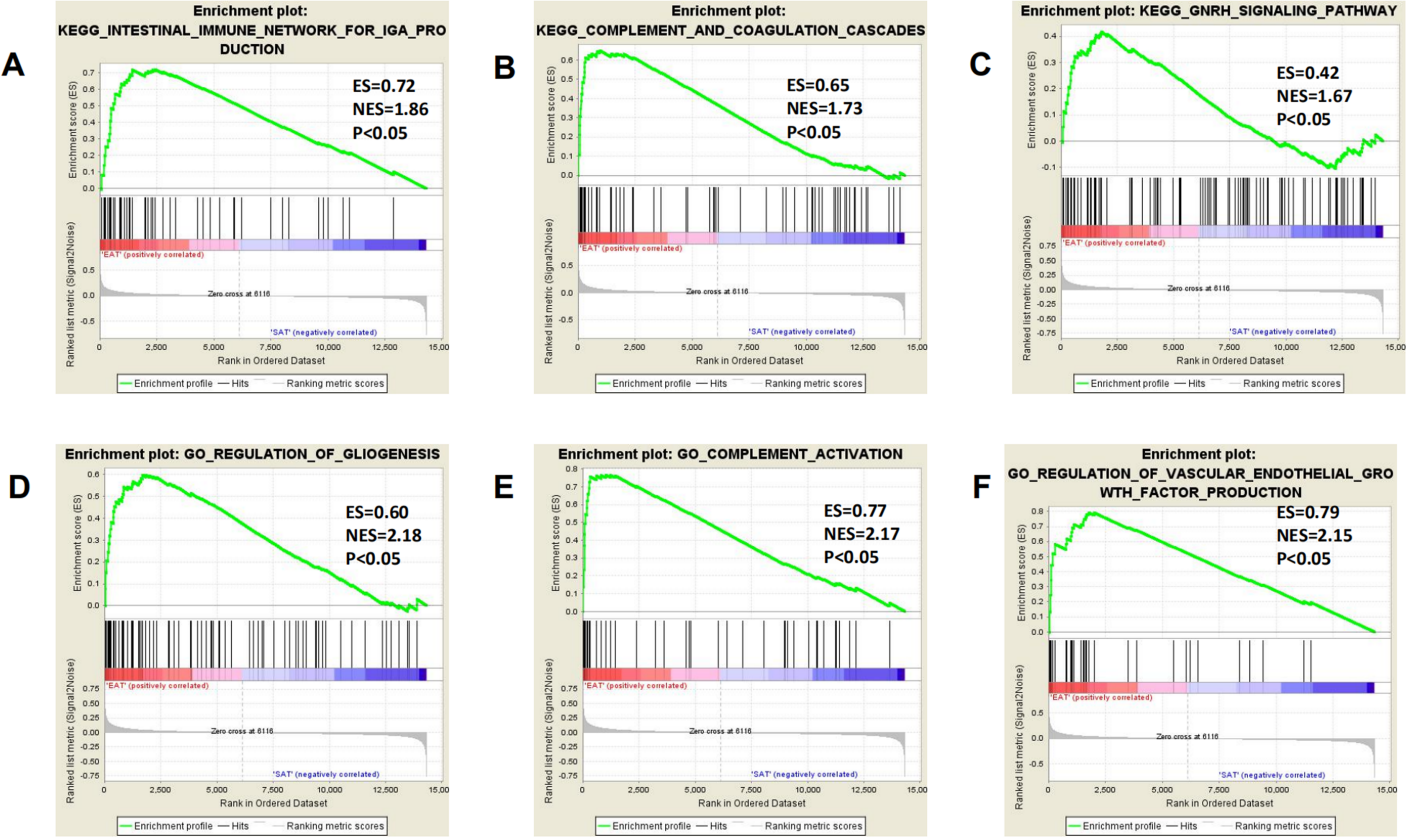
Three representitive new enriched pathways and three gene sets in the EAT group from GSEA analysis. GSEA, gene set enrichment analysis; ES, enrichment score; NES, normalised enrichment score. (A) KEGG: intestinal immune network for IgA production; (B) KEGG: complement and coagulation cascades; (C) KEGG: GNRH signaling pathway; (D) GO: regulation of gliogenesis; (E) GO: complement activation; (F) GO: vascular endothelial growth factor production regulation.

All expression data were submitted to GSEA to test and verify the results of KEGG and GO analysis and to excavate new EAT functions. The samples were divided into EAT and SAT group and subjected to GSEA. The GSEA results showed high repeatability and accuracy compared to KEGG and GO analysis. These results showed high similarity with KEGG and GO analysis, including the enriched gene set CCR chemokine receptor binding and the enriched pathways prion diseases and chemokine signalling pathway supply. Moreover, genes and functions were mostly enriched in chemokine up-regulation, immune cell activation, and lipid metabolism, which was shown by several EAT studies. Additionally, we noted some rarely seen enriched functions. These included the KEGG pathways intestinal immune network for IgA production and complement and coagulation cascades and the gene sets gliogenesis regulation, complement activation, and vascular endothelial growth factor production regulation (Fig. 4). These EAT functions were rarely discussed in past studies, and they could be new directions for EAT studies. The top 20 results were listed in Tables S2 and S3.

### PPI network construction and analysis

**Figure 5:**
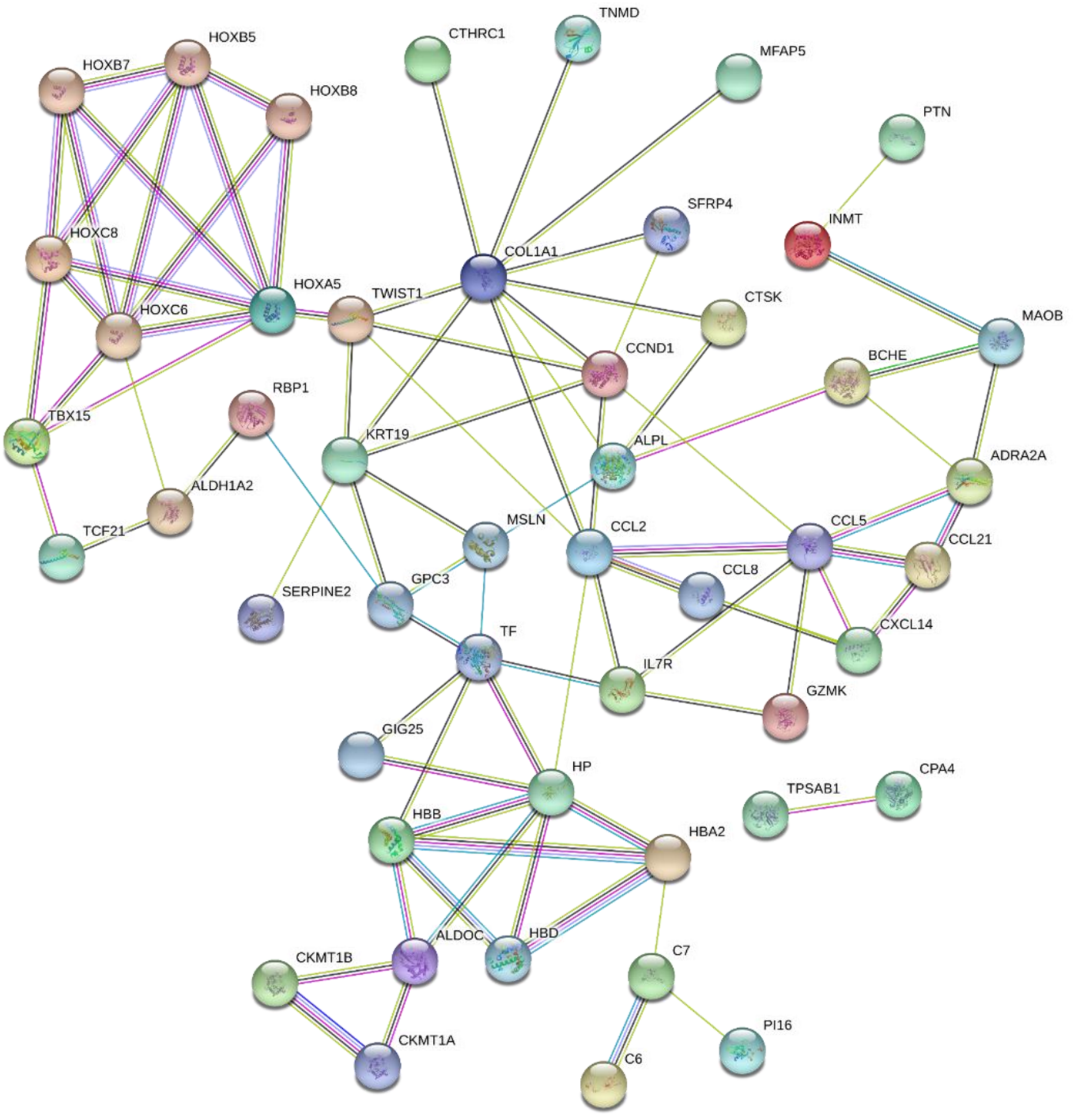
PPI network of network of proteins constructed by the DEGs. PPI network included 86 nodes and 87 edges. Medium confidnce = 0.400. PPI, protein–protein interaction.

**Figure 6:**
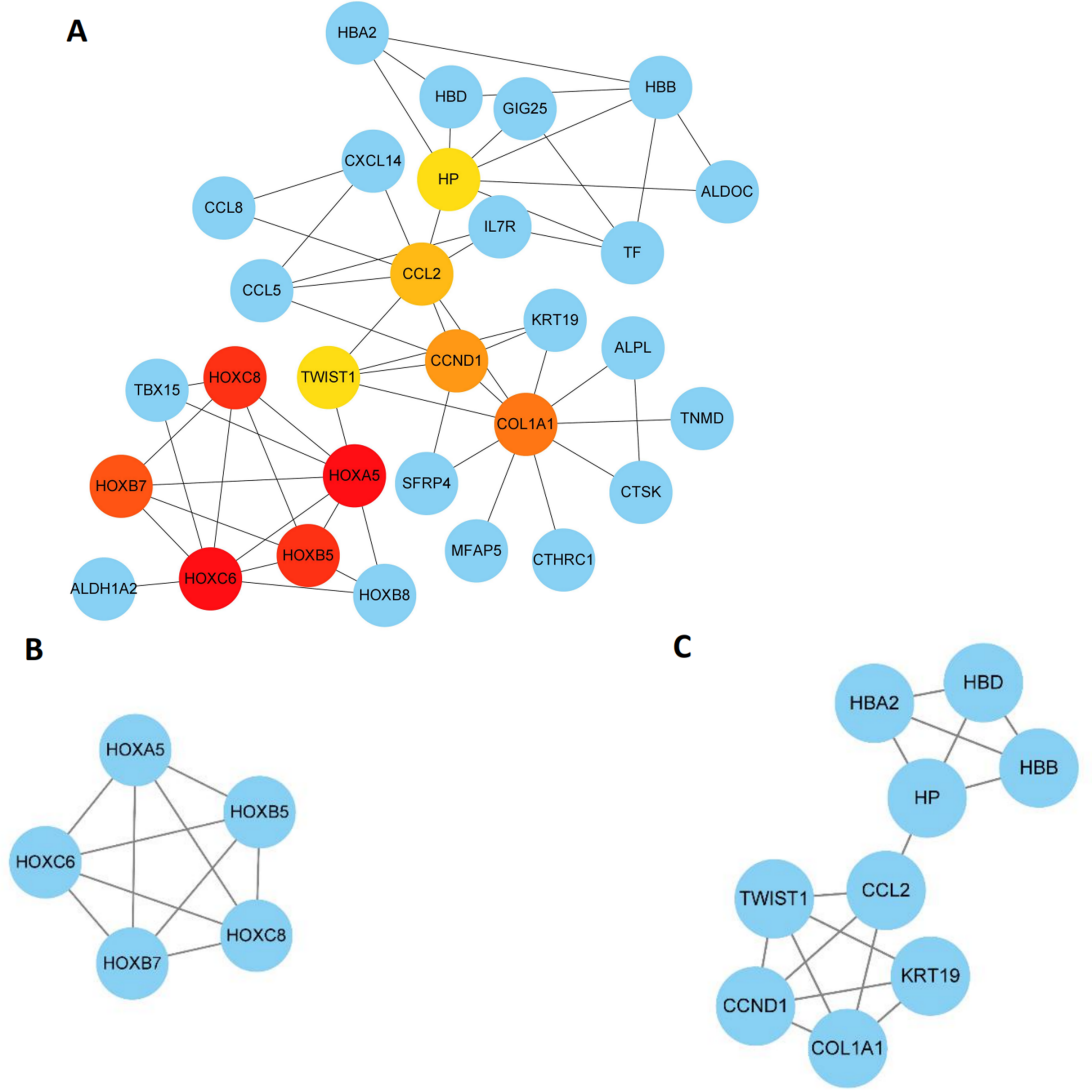
Key modules and hub genes identified in cytoscape. (A) 10 Hub genes identified by CytoHubba, (B) and (C) 2 key modules identified by MCODE in Cytoscape.

The 89 DEGs were submitted to the STRING database to predict the interactions between proteins. The PPI network of DEGs was constructed using a combined score greater than 0.4 (Fig. 5), and the two most significant modules were obtained using MCODE in Cytoscape (Fig. 6B). The first module included homeobox B5 (HOXB5), homeobox (HOXC8), homeobox C6 (HOXC6), homeobox A5 (HOXA5) and homeobox B7 (HOXB7). The second module consisted of haemoglobin subunit delta (HBD), haemoglobin subunit beta (HBB), haemoglobin subunit alpha 2 (HBA2), haptoglobin (HP), C–C motif chemokine ligand 2 (CCL2), cyclin D1 (CCND1), Thy-1 cell surface antigen (HP), collagen type I alpha 1 chain (COL1A1), twist family bHLH transcription factor 1 (TWIST1), and keratin 19 (KRT19) (Fig. 6C). The hub genes selected from the PPI network using the MCC algorithm and cytoHubba plugin were shown in Fig. 6A. The top 10 hub genes identified by MCC are HOXA5, HOXC6, HOXC8, HOXB5, HOXB7, COL1A1, CCND1, CCL2, HP and TWIST1 (Fig. 6A).

### Regulating miRNA prediction

The top 10 hub genes from Cytoscape were submitted to online tool mirDIP. The top five predicted miRNAs of these genes were chosen and listed in Table 3. Among these miRNAs, hsa-miR-196a-5p and hsa-miR-196b-5p could play more essential roles in CAD pathogenesis.

**Table 3:** Top 5 predicted miRNAs of 10 hub genes predicted by DEGs. Top 10 hub genes were submitted to online tool mirDIP, then top 5 predicted miRNAs of these genes were outputted and listed.

Genes	Predicted miRNAs
HOXA5	hsa-miR-26b-5p, hsa-miR-26a-5p, hsa-miR-196a-5p, hsa-miR-196b-5p, hsa-miR-96-5p
HOXB5	hsa-miR-23b-3p, hsa-miR-181c-5p, hsa-miR-181a-5p, hsa-miR-181b-5p, hsa-miR-23a-3p
HOXC6	hsa-miR-27a-3p, hsa-miR-27b-3p, hsa-miR-377-3p, hsa-miR-128-3p, hsa-miR-574-5p
HOXC8	hsa-miR-196a-5p, hsa-miR-196b-5p, hsa-miR-152-3p, hsa-miR-148a-3p, hsa-miR-148b-3p
HOXB7	hsa-miR-196a-5p, hsa-miR-196b-5p, hsa-miR-195-5p, hsa-miR-376c-3p, hsa-miR-524-5p
COL1A1	hsa-miR-29a-3p, hsa-miR-29c-3p, hsa-let-7c-5p, hsa-let-7c-5p, hsa-let-7a-5p
CCND1	hsa-miR-195-5p, hsa-miR-16-5p, hsa-miR-15b-5p, hsa-miR-15a-5p, hsa-miR-106b-5p
CCL2	hsa-miR-374a-5p, hsa-miR-374b-5p, hsa-miR-1-3p, hsa-miR-206, hsa-miR-613
HP	
TWIST1	hsa-miR-151a-3p, hsa-miR-137, hsa-miR-361-5p, hsa-miR-96-5p, hsa-miR-32-5p

### RT-qPCR validation of mRNA expression of top 10 hub genes

**Figure 7:**
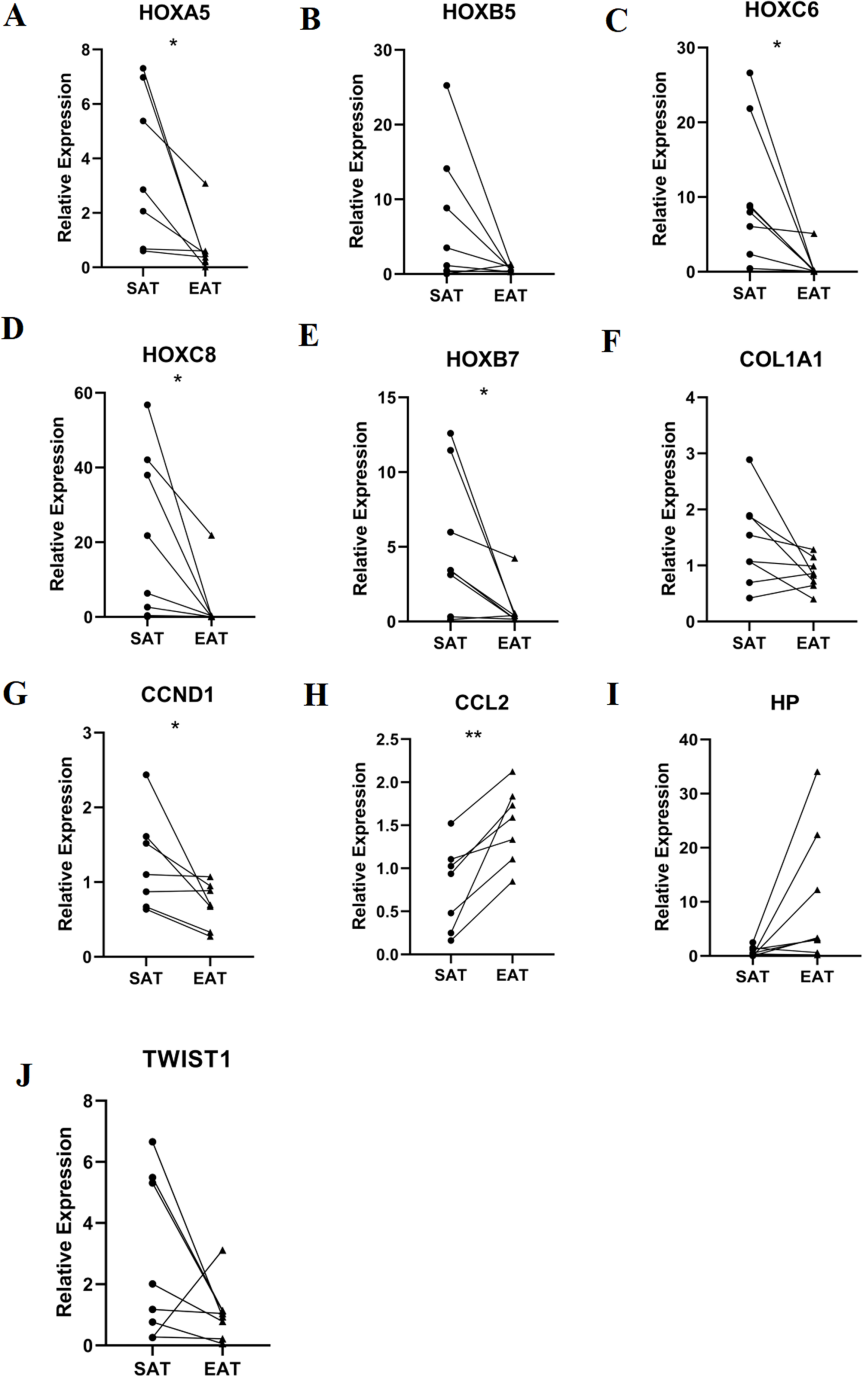
Relative expression of top 10 hub genes. Including (A) HOXA5, (B) HOXB5, (C) HOXC6, (D) HOXC8, (E) HOXB7, (F) COL1A1, (G) CCND1, (H) CCL2, (I) HP and (J) TWIST1 measured by real time-qPCR. *P < 0.05; **P < 0.01.

RT-qPCR was performed using the total RNA extracted from eight pairs of EAT and SAT from patients with CAD undergoing CABG, which were used to confirm the expression levels of 10 hub genes obtained from Cytoscape. The RT-qPCR experiment results showed a significant change in the expression of six hub genes, including HOXA5, HOXC6, HOXC8, HOXB7, CCND1 and CCL2 in Fig. 7 (*P* value < 0.05). The mean and standard deviation of data was listed in Table 4.

**Table 4:** Mean and standard deviation and *P* value of relative expression of RT-PCR.

Gene	SAT	EAT	*P* value
HOXA5	3.69 ± 2.85	0.71 ± 1.06	0.034
HOXC6	10.35 ± 9.16	0.74 ± 1.77	0.026
HOXC8	20.99 ± 22.13	2.86 ± 7.69	0.04
HOXB5	6.70 ± 9.01	0.7 ± 4.17	0.097
HOXB7	5.06 ± 4.70	0.81 ± 1.39	0.038
COL1A1	1.43 ± 0.79	0.86 ± 0.28	0.073
CCND1	1.26 ± 0.64	0.69 ± 0.30	0.049
CCL2	0.78 ± 0.50	1.51 ± 0.44	0.004
HP	0.76 ± 0.88	9.86 ± 12.31	0.069
TWIST1	2.74 ± 2.64	1.05 ± 0.93	0.137

## Discussion

Epicardial adipose tissue, adipose tissue pools in the vicinity of blood vessels and myocardium, are one of the critical factors of CAD (Iacobellis, Barbaro & Gerstein, 2008; Cetin et al., 2013). The effect of EAT paracrine and vasocrine regulation of the atherosclerosis process via the proinflammatory response is widely recognised (Alexopoulos, Katritsis & Raggi, 2014). However, limited research findings cannot fully reveal the complete mechanism of epicardial fat’s effect on CAD. The recent rapid advance in microarray analysis has greatly contributed to understanding EAT expression profile alterations in CAD. However, few studies have integrated these datasets together.

A past expression analysis showed genes related to CAD (C6, CXCR4, CBS and CNTNAP2) (Guauque-Olarte et al., 2011). Another EAT profiling revealed that EAT in CAD patients had up-regulated pro-inflammatory pathways, macrophage surface antigens, and cytokines (Vacca et al., 2016). A previous transcriptome analysis described different characteristics between EAT and SAT, and it showed overexpressed gene characteristics of the ‘beige’/‘brite’ phenotypes (Gaborit et al., 2015). Another results showed ADORA1, adiponectin, AGT, ADM, CATA, IL-1β, MCP-1, RBP-4, TNF-α and UCP-1 may play significant roles in the unique physiology of EAT and/or its role in pathophysiology, through mechanisms as diverse as steroid hormone responses and regulation of apoptosis (Yim & Rabkin, 2017). Our results showed high similarity with these findings including the overexpression of proinflammatory factors like C6 and CCL2. However, some new possible function of EAT were predicted by our analysis.

Finally, 89 DEGs in several possible gene sets and pathways were screened and identified as highly enriched in CAD patient EAT samples. GO and KEGG analysis showed that DEGs were mainly associated biological processes like G protein-coupled receptor binding, chemokine activity, chemokine receptor binding, and retinoid binding. The GSEA results from all expression data showed high similarity with DEG functional analysis, most of which are closely related to CAD genes and progression. Remarkably, pro-inflammatory and immunological genes and pathways increased, including CCR chemokine receptor binding and lymphocyte mediated immunity, which supposed that inflammatory factors and immune cell activation may play an essential role in regulating the CAD process. Moreover, GSEA analysis discovered some seldom seen enriched functions, including the KEGG pathways intestinal immune network for IgA production and complement and coagulation cascades and the gene sets gliogenesis regulation, complement activation, and vascular endothelial growth factor production regulation. These EAT results suggested that more attention should be paid to these functions of EAT including interaction with the intestinal immune system, complement activation, and extracellular matrix regulation.

A DEG PPI network was constructed. Two key modules and 10 hub genes were identified, including HOXA5, HOXB5, HOXC6, HOXC8, HOXB7, COL1A1, CCND1, CCL2, HP and TWIST1. Moreover, we validated the results using RT-PCR with eight pairs of samples from CAD patients.

Hox genes encode for highly conserved homeodomain transcription factors. Some members of the HOX family have been participating in vascular remodelling, angiogenesis, and diseases associated with changes in matrix degradation, integrins, and extracellular matrix components (Gorski & Walsh, 2000). The HOX family members exhibit a high self-interaction level, including forming chromatin conformations known as topological domains (Dixon et al., 2012). HOXA5 protein transduction domain overexpression prevents inflammation as shown by inhibition of TNFα-inducible monocyte binding to HUVECs (Lee et al., 2011; Chen & Gorski, 2008). HOXB5 up-regulation could enhance blood vessel perfusion in vivo by increasing MCP-1 and IL-6 expression and enhancing leucocyte infiltration and blood vessel remodelling in ischaemic disease (Fessner et al., 2014). CCND1 is a critical protein in the cell cycle, and cell cycle disorder plays an important role in ventricular remodelling pathogenesis (Chandrashekhar, 2005; Abbate, Biondi-Zoccai & Baldi, 2002). A previous study indicated that dysregulated cardiomyocyte cell cycle progression played a role in left ventricle remodelling and dysfunction in dilated cardiomyopathy (Ibe et al., 2007). CCL2 is an important inflammatory cytokine participating in the CAD pathological process. A 2-year survival analysis showed that low CCL2 serum levels was highly associated with myocardial infarction (Leocadio et al., 2019). CCL2 was also reportedly involved in human atherosclerosis and myocardial infarction pathogenesis (McDermott et al., 2005). TWIST expression could promote developmental angiogenesis by inducing EC proliferation and migration. In addition to its role in development, a recent study showed that TWIST is highly expressed at low shear stress regions of adult arteries where it plays a role in promoting atherosclerosis by inducing EC proliferation and inflammation (Mahmoud et al., 2016). HP is an acute phase protein that is always overexpressed during inflammation. A clinical study showed that plasma HP concentrations are increased in patients with CAD and were closely associated with luminal stenosis severity (Lee et al., 2013).

In addition to hub genes discussed above, the other four genes have rarely been studied in CAD. HOXC6, HOXC8 and HOXB7 have been shown to be prognostic biomarkers and therapeutic targets in cancer research, including prostate cancer (Van Neste et al., 2016; Axlund, Lambert & Nordeen, 2010), gastric cancer (Chen et al., 2016), oesophageal squamous cell carcinoma (Du et al., 2014), nasopharyngeal carcinoma (Jiang et al., 2015), and acute lymphoblastic leukaemia (Zhong et al., 2019). In our bioinformatic and PCR results, most HOX genes were identified downregulated in the EAT from CAD patients. While recent studies have revealed the role of HOX proteins in heart development, little is known about the function in CAD development. However, due to the important function of HOX in regulating various signalling pathways including Wnt, TGF-β, MAPK, PI3K/Akt and NF-kB (Yu, Zhan & Zhang, 2020), we assume that the downregulation of HOX genes could inhibited the transcription of protective adipocytokines. In this way, HOX genes could play critical roles in the pathophysiological process of CAD. COL1A1 encodes the pro-alpha 1 chains of type I collagen. Type I is a fibril-forming collagen that is found in most connective tissues like bone, cornea, dermis and tendon. A new report showed that COL1A1 gene polymorphisms are associated with heart morphology changes and myocardium and vessel relaxation process deflection (Nekhanevych et al., 2018). In addition, COL1A1 genes were also identified downregulated in the EAT of CAD patients in our study. A new study reported the upregulation of the COL1A1 could be a plasma biomarker of CAD (LeungOng et al., 2020). Type I collagen is a critical part in the extracellular matrix of myocardium. Efficient deposition of type I collagen is fundamental to healing injured myocardium. The downregulation in EAT and the upregulation in plasma showed the degradation of type I collagen and the release of COL1A1 to the blood could be a important pathological process in CAD.

Finally, several miRNAs were predicted using online tool. Among these miRNAs, hsa-miR-196a-5p and hsa-miR-196b-5p garnered the most attention. However, these two miRNAs have not been studied in the CAD field, so they need more exploration.

Here, we merged three expression profiles to explore some new molecular characteristics in EAT from CAD patients. The sva package was used to eliminate batch effects. Our study found some new common DEGs and enriched gene sets and pathways based on more samples compared to a previous study. In addition, we analysed all expression data rather than DEGs with GSEA software to excavate EAT features from a comprehensive perspective.

However, several limitations are associated with our study. In our study, SAT samples were chosen as control groups rather than EAT from healthy people for following reasons: First, healthy EAT controls is hard to acquire due to ethical limitations. Also, the baseline data downloaded from the GEO data sets were incomplete since most EAT control samples were obtained from other patients without CAD. Therefore, SAT controls were relatively reliable and used to eliminate baseline bias. In addition, We still do not know if EAT and SAT differ in healthy people.

Here, we discovered clues related to genes and pathways using bioinformatic analysis. These results could help us excavate key biomarkers and EAT targets in CAD. This could provide more points to explore CAD pathogenesis. Next, we plan to experimentally verify these genes and pathways in human EAT samples. We will explore them in depth, so that we can further reveal EAT’s important role in CAD pathogenesis.

## Conclusions

Here, we used comprehensive bioinformatic analysis to identify and functionally analyse the DEGs between EAT and SAT in CAD patients. We revealed EAT could participating the CAD through DEGs including HOXA5, HOXB5, HOXC6, HOXC8, HOXB7, COL1A1, CCND1, CCL2, HP and TWIST1; as well as some new pathways including interaction with the intestinal immune system, complement activation, and extracellular matrix regulation. RT-PCR was used to validate the bioinformatic results. Some miRNAs regulating these key genes were predicted. Some of our results are similar to other studies, but some new conclusions were also drawn. These results could help us to explore EAT’s role in CAD from new and in-depth perspectives.

## Supplemental Information

10.7717/peerj.8763/supp-1 Supplemental Information 1 primers of RT-PCR.

10.7717/peerj.8763/supp-2 Supplemental Information 2 The distribution of raw data and standardised data in GSE24425, GSE64554 and GSE120774. The *Y* axis is the log2 of the expression of each gene.

10.7717/peerj.8763/supp-3 Supplemental Information 3 All significant GO pathways enriched by DEGs.

10.7717/peerj.8763/supp-4 Supplemental Information 4 Top 20 significant KEGG pathways enriched by DEGs in GSEA.

10.7717/peerj.8763/supp-5 Supplemental Information 5 Top 20 significant GO pathways enriched by DEGs in GSEA.

10.7717/peerj.8763/supp-6 Supplemental Information 6 Row probedata and annotation of GSE120774.

10.7717/peerj.8763/supp-7 Supplemental Information 7 Row probedata and annotation of GSE64554.

10.7717/peerj.8763/supp-8 Supplemental Information 8 Row probedata and annotation of GSE24425.

10.7717/peerj.8763/supp-9 Supplemental Information 9 Results index from GSEA.

10.7717/peerj.8763/supp-10 Supplemental Information 10 Raw data of RT-PCR.

10.7717/peerj.8763/supp-11 Supplemental Information 11 All code of R and Perl.

## Additional Information and Declarations

### Data Availability

The following information was supplied regarding data availability: The raw code and raw data are available in the Supplemental Files.

## References

[ref-1] Abbate A, Biondi-Zoccai GG, Baldi A (2002). Pathophysiologic role of myocardial apoptosis in post-infarction left ventricular remodeling. Journal of Cellular Physiology.

[ref-2] Alexopoulos N, Katritsis D, Raggi P (2014). Visceral adipose tissue as a source of inflammation and promoter of atherosclerosis. Atherosclerosis.

[ref-3] Axlund SD, Lambert JR, Nordeen SK (2010). HOXC8 inhibits androgen receptor signaling in human prostate cancer cells by inhibiting SRC-3 recruitment to direct androgen target genes. Molecular Cancer Research.

[ref-4] Bader GD, Hogue CW (2003). An automated method for finding molecular complexes in large protein interaction networks. BMC Bioinformatics.

[ref-5] Cetin M, Cakici M, Polat M, Suner A, Zencir C, Ardic I (2013). Relation of epicardial fat thickness with carotid intima-media thickness in patients with type 2 diabetes mellitus. International Journal of Endocrinology.

[ref-6] Chandrashekhar Y (2005). Role of apoptosis in ventricular remodeling. Current Heart Failure Reports.

[ref-7] Chen Y, Gorski DH (2008). Regulation of angiogenesis through a microRNA (miR-130a) that down-regulates antiangiogenic homeobox genes GAX and HOXA5. Blood.

[ref-8] Chen S-W, Zhang Q, Xu Z-F, Wang H-P, Shi Y, Xu F, Zhang W-J, Wang P, Li Y (2016). HOXC6 promotes gastric cancer cell invasion by upregulating the expression of MMP9. Molecular Medicine Reports.

[ref-9] Chin C-H, Chen S-H, Wu H-H, Ho C-W, Ko M-T, Lin C-Y (2014). cytoHubba: identifying hub objects and sub-networks from complex interactome. BMC Systems Biology.

[ref-10] Dixon JR, Selvaraj S, Yue F, Kim A, Li Y, Shen Y, Hu M, Liu JS, Ren B (2012). Topological domains in mammalian genomes identified by analysis of chromatin interactions. Nature.

[ref-11] Du Y-B, Dong B, Shen L-Y, Yan W-P, Dai L, Xiong H-C, Liang Z, Kang X-Z, Qin B, Chen K-N (2014). The survival predictive significance of HOXC6 and HOXC8 in esophageal squamous cell carcinoma. Journal of Surgical Research.

[ref-12] Fessner A, Esser JS, Bluhm F, Grundmann S, Zhou Q, Patterson C, Bode C, Moser M (2014). The transcription factor HoxB5 stimulates vascular remodelling in a cytokine-dependent manner. Cardiovascular Research.

[ref-13] Gaborit B, Venteclef N, Ancel P, Pelloux V, Gariboldi V, Leprince P, Amour J, Hatem SN, Jouve E, Dutour A, Clément K (2015). Human epicardial adipose tissue has a specific transcriptomic signature depending on its anatomical peri-atrial, peri-ventricular, or peri-coronary location. Cardiovascular Research.

[ref-14] Ginestet C (2011). ggplot2: elegant graphics for data analysis. Journal of the Royal Statistical Society Series A: Statistics in Society.

[ref-15] Gorski DH, Walsh K (2000). The role of homeobox genes in vascular remodeling and angiogenesis. Circulation Research.

[ref-16] Gruzdeva OV, Akbasheva OE, Dyleva YA, Antonova LV, Matveeva VG, Uchasova EG, Fanaskova EV, Karetnikova VN, Ivanov SV, Barbarash OL (2017). Adipokine and cytokine profiles of epicardial and subcutaneous adipose tissue in patients with coronary heart disease. Bulletin of Experimental Biology and Medicine.

[ref-17] Guauque-Olarte S, Gaudreault N, Piche ME, Fournier D, Mauriège P, Mathieu P, Bossé Y (2011). The transcriptome of human epicardial, mediastinal and subcutaneous adipose tissues in men with coronary artery disease. PLOS ONE.

[ref-18] Herrington W, Lacey B, Sherliker P, Armitage J, Lewington S (2016). Epidemiology of atherosclerosis and the potential to reduce the global burden of atherothrombotic disease. Circulation Research.

[ref-19] Hirata Y, Tabata M, Kurobe H, Motoki T, Akaike M, Nishio C, Higashida M, Mikasa H, Nakaya Y, Takanashi S, Igarashi T, Kitagawa T, Sata M (2011). Coronary atherosclerosis is associated with macrophage polarization in epicardial adipose tissue. Journal of the American College of Cardiology.

[ref-20] Huang D, Sherman BT, Tan Q, Collins JR, Alvord WG, Roayaei J, Stephens R, Baseler MW, Lane HC, Lempicki RA (2007). The DAVID gene functional classification tool: a novel biological module-centric algorithm to functionally analyze large gene lists. Genome Biology.

[ref-21] Iacobellis G, Barbaro G, Gerstein HC (2008). Relationship of epicardial fat thickness and fasting glucose. International Journal of Cardiology.

[ref-22] Ibe W, Saraste A, Lindemann S, Bruder S, Buerke M, Darius H, Pulkki K, Voipio-Pulkki L-M (2007). Cardiomyocyte apoptosis is related to left ventricular dysfunction and remodelling in dilated cardiomyopathy, but is not affected by growth hormone treatment. European Journal of Heart Failure.

[ref-23] Jia S-J, Niu P-P, Cong J-Z, Zhang B-K, Zhao M (2014). TLR4 signaling: a potential therapeutic target in ischemic coronary artery disease. International Immunopharmacology.

[ref-24] Jiang Y, Yan B, Lai W, Shi Y, Xiao D, Jia J, Liu S, Li H, Lu J, Li Z, Chen L, Chen X, Sun L, Muegge K, Cao Y, Tao Y (2015). Repression of Hox genes by LMP1 in nasopharyngeal carcinoma and modulation of glycolytic pathway genes by HoxC8. Oncogene.

[ref-25] Kohl M, Wiese S, Warscheid B (2011). Cytoscape: software for visualization and analysis of biological networks. Methods in Molecular Biology.

[ref-26] Kolde R (2015). Pheatmap: pretty heatmaps. https://cran.r-project.org/web/packages/pheatmap/index.html.

[ref-27] Lee C-W, Cheng T-M, Lin C-P, Pan J-P (2013). Plasma haptoglobin concentrations are elevated in patients with coronary artery disease. PLOS ONE.

[ref-28] Lee JY, Park KS, Cho EJ, Joo HK, Lee SK, Lee SD, Park JB, Chang SJ, Jeon BH (2011). Human HOXA5 homeodomain enhances protein transduction and its application to vascular inflammation. Biochemical and Biophysical Research Communications.

[ref-29] Leek JT, Johnson WE, Parker HS, Jaffe AE, Storey JD (2012). The sva package for removing batch effects and other unwanted variation in high-throughput experiments. Bioinformatics.

[ref-30] Leocadio PCL, Dos Reis Menta PL, Dias MTS, Fraga JR, Goulart AC, Santos IS, Lotufo PA, Bensenor IM, Alvarez-Leite JI (2019). Low serum levels of CCL2 are associated with worse prognosis in patients with acute coronary syndrome: 2-year survival analysis. Biomedicine & Pharmacotherapy.

[ref-31] LeungOng K, Shan Chung RW, Hui N, Festin K, Lundberg AK, Rye K-A, Jonasson L, Kristenson M (2020). Usefulness of certain protein biomarkers for prediction of coronary heart disease. American Journal of Cardiology.

[ref-32] Liberzon A, Subramanian A, Pinchback R, Thorvaldsdottir H, Tamayo P, Mesirov JP (2011). Molecular signatures database (MSigDB) 3.0. Bioinformatics.

[ref-33] Liu Z, Wang S, Wang Y, Zhou N, Shu J, Stamm C, Jiang M, Luo F (2019). Association of epicardial adipose tissue attenuation with coronary atherosclerosis in patients with a high risk of coronary artery disease. Atherosclerosis.

[ref-34] Mahabadi AA, Berg MH, Lehmann N, Kälsch H, Bauer M, Kara K, Dragano N, Moebus S, Jöckel K-H, Erbel R, Möhlenkamp S (2013). Association of epicardial fat with cardiovascular risk factors and incident myocardial infarction in the general population: the Heinz Nixdorf recall study. Journal of the American College of Cardiology.

[ref-35] Mahmoud MM, Kim HR, Xing R, Hsiao S, Mammoto A, Chen J, Serbanovic-Canic J, Feng S, Bowden NP, Maguire R, Ariaans M, Francis SE, Weinberg PD, Van Der Heiden K, Jones EA, Chico TJA, Ridger V, Evans PC (2016). TWIST1 integrates endothelial responses to flow in vascular dysfunction and atherosclerosis. Circulation Research.

[ref-36] McDermott DH, Yang Q, Kathiresan S, Cupples LA, Massaro JM, Keaney JF, Larson MG, Vasan RS, Hirschhorn JN, O’Donnell CJ, Murphy PM, Benjamin EJ (2005). CCL2 polymorphisms are associated with serum monocyte chemoattractant protein-1 levels and myocardial infarction in the Framingham heart study. Circulation.

[ref-37] Nakanishi K, Fukuda S, Tanaka A, Otsuka K, Jissho S, Taguchi H, Yoshikawa J, Shimada K (2014). Persistent epicardial adipose tissue accumulation is associated with coronary plaque vulnerability and future acute coronary syndrome in non-obese subjects with coronary artery disease. Atherosclerosis.

[ref-38] Nekhanevych О, Zhyliuk V, Logvinenko V, Onul N, Khomiak O, Jissho S, Taguchi H, Yoshikawa J, Shimada K (2018). Cardiovascular system and musculoskeletal changes of the sportsmen with polymorphisms of COL1A1 gene. Georgian Medical News.

[ref-39] Niu J, Kolattukudy PE (2009). Role of MCP-1 in cardiovascular disease: molecular mechanisms and clinical implications. Clinical Science.

[ref-40] Ouchi N, Walsh K (2008). A novel role for adiponectin in the regulation of inflammation. Arteriosclerosis, Thrombosis, and Vascular Biology.

[ref-41] Patel VB, Mori J, McLean BA, Basu R, Das SK, Ramprasath T, Parajuli N, Penninger JM, Grant MB, Lopaschuk GD, Oudit GY (2016). ACE2 deficiency worsens epicardial adipose tissue inflammation and cardiac dysfunction in response to diet-induced obesity. Diabetes.

[ref-42] R Development Core Team (2018). R: a language and environment for statistical computing.

[ref-43] Salgado-Somoza A, Teijeira-Fernandez E, Luis Fernandez A, González-Juanatey JR, Eiras S (2010). Proteomic analysis of epicardial and subcutaneous adipose tissue reveals differences in proteins involved in oxidative stress. American Journal of Physiology-Heart and Circulatory Physiology.

[ref-44] Subramanian A, Tamayo P, Mootha VK, Mukherjee S, Ebert BL, Gillette MA, Paulovich A, Pomeroy SL, Golub TR, Lander ES, Mesirov JP (2005). Gene set enrichment analysis: a knowledge-based approach for interpreting genome-wide expression profiles. Proceedings of the National Academy of Sciences of the United States of America.

[ref-45] Szklarczyk D, Franceschini A, Wyder S, Forslund K, Heller D, Huerta-Cepas J, Simonovic M, Roth A, Santos A, Tsafou KP, Kuhn M, Bork P, Jensen LJ, Von Mering C (2015). STRING v10: protein–protein interaction networks, integrated over the tree of life. Nucleic Acids Research.

[ref-46] Thomou T, Mori MA, Dreyfuss JM, Konishi M, Sakaguchi M, Wolfrum C, Rao TN, Winnay JN, Garcia-Martin R, Grinspoon SK, Gorden P, Kahn CR (2017). Adipose-derived circulating miRNAs regulate gene expression in other tissues. Nature.

[ref-47] Tokar T, Pastrello C, Rossos AEM, Abovsky M, Hauschild A-C, Tsay M, Lu R, Jurisica I (2018). mirDIP 4.1-integrative database of human microRNA target predictions. Nucleic Acids Research.

[ref-48] Vacca M, Di Eusanio M, Cariello M, Graziano G, D’Amore S, Petridis FD, D’orazio A, Salvatore L, Tamburro A, Folesani G, Rutigliano D, Pellegrini F, Sabbà C, Palasciano G, Di Bartolomeo R, Moschetta A (2016). Integrative miRNA and whole-genome analyses of epicardial adipose tissue in patients with coronary atherosclerosis. Cardiovascular Research.

[ref-49] Van Neste L, Hendriks RJ, Dijkstra S, Trooskens G, Cornel EB, Jannink SA, De Jong H, Hessels D, Smit FP, Melchers WJG, Leyten GHJM, De Reijke TM, Vergunst H, Kil P, Knipscheer BC, Van De Kaa CAH, Mulders PFA, Van Oort IM, Van Criekinge W, Schalken JA (2016). Detection of high-grade prostate cancer using a urinary molecular biomarker-based risk score. European Urology.

[ref-50] Wu Z-H, Zhao S-P (2006). Adipocyte: a potential target for the treatment of atherosclerosis. Medical Hypotheses.

[ref-51] Yao F, Lv YC, Zhang M, Xie W, Tan Y-L, Gong D, Cheng H-P, Liu D, Li L, Liu X-Y, Zheng X-L, Tang C-K (2015). Apelin-13 impedes foam cell formation by activating class III PI3K/Beclin-1-mediated autophagic pathway. Biochemical and Biophysical Research Communications.

[ref-52] Yim J, Rabkin SW (2017). Differences in Gene Expression and Gene Associations in Epicardial fat compared to subcutaneous fat. Hormone and Metabolic Research.

[ref-53] Yu G, Wang L, Han Y, He Q-Y (2012). clusterProfiler: an R package for comparing biological themes among gene clusters. OMICS: A Journal of Integrative Biology.

[ref-54] Yu M, Zhan J, Zhang H (2020). HOX family transcription factors: related signaling pathways and post-translational modifications in cancer. Cellular Signalling.

[ref-55] Zhang BK, Lai X, Jia SJ (2015). Epigenetics in atherosclerosis: a clinical perspective. Discovery Medicine.

[ref-56] Zhong Y, Zhang Y, Ma D, Ren X, Xu C, Wan D (2019). Inhibition of HOXB7 suppresses p27-mediated acute lymphoblastic leukemia by regulating basic fibroblast growth factor and ERK1/2. Life Sciences.

