# Supplementary material for: Identification of key genes and pathways affected in epicardial adipose tissue from patients with coronary artery disease by integrated bioinformatics analysis
Source: PeerJ. 2020 Mar 25;8:e8763. doi: 10.7717/peerj.8763 (PMC7102503; doi:10.7717/peerj.8763)
Supplement: Supplemental Information 1 [file peerj-08-8763-s001.docx]

HOXA5 gene: 5’- TCTCGTTGCCCTAATTCATCTTTT -3’ (Forward)

5’- CATTCAGGACAAAGAGATGAACAGAA -3’ (Reverse).

HOXB5 gene: 5’- CAATGGGATGGACCTCAGCGTC -3’ (Forward)

5’- CCCGGTCATATCATGGCTGATG-3’ (Reverse).

HOXC6 gene: 5’- ACAGACCTCAATCGCTCAGGA -3’ (Forward)

5’- AGGGGTAAATCTGGATACTGGC -3’ (Reverse).

HOXC8 gene: 5’- CGCACCACGTTCAAGACTTCT -3’ (Forward)

5’- TAAGCGAGCACGGGTTCTG -3’ (Reverse).

HOXB7 gene: 5’- ATCTACCCCTGGATGCGAAGCT -3’ (Forward)

5’- GCGTCAGGTAGCGATTGTAGTG -3’ (Reverse).

TWIST1 gene: 5’- AGCTACGCCTTCTCGGTCT -3’ (Forward)

5’- CCTTCTCTGGAAACAATGACATC -3’ (Reverse).

CCL2 gene: 5’- GCGGAGCTATAGAAGAATCAC -3’ (Forward)

5’- TTGGGTTGTGGAGTGAGTGT -3’ (Reverse).

HP gene: 5’- GCAAGACCAACCAAGATG -3’ (Forward)

5’- CATCATTGCCTGAGTCCACT -3’ (Reverse).

CCND1 gene: 5’- TCCTCTCCAAAATGCCAGAG -3’ (Forward)

5’- GGCGGATTGGAAATGAACTT -3’ (Reverse).

COL1A1 gene: 5’- GCTTCACCTACAGCGTCACTGTCG -3’ (Forward)

5’- AGAGGAGTTTACAGGAAGCAGACAG -3’ (Reverse).

β-actin gene: 5’- TGGACTTCGAGCAAGAGATG -3’ (Forward)

5’- TGTTGGCGTACAGGTCTTTG -3’ (Reverse).
