# Supplementary figures and images for: Identification of key genes and pathways affected in epicardial adipose tissue from patients with coronary artery disease by integrated bioinformatics analysis
Source: PeerJ. 2020 Mar 25;8:e8763. doi: 10.7717/peerj.8763 (PMC7102503; doi:10.7717/peerj.8763)

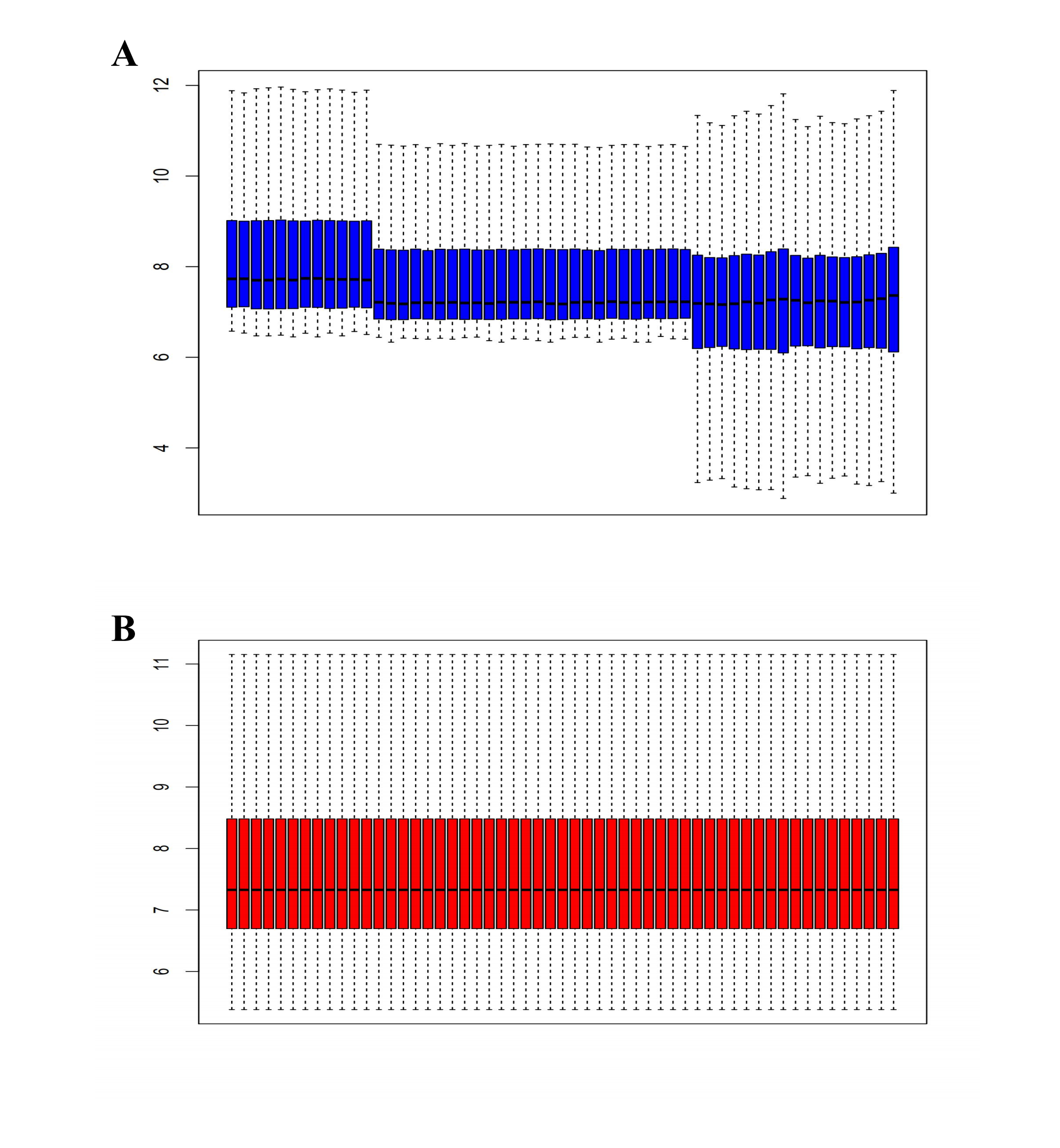

Supplement: Supplemental Information 2 — The Y axis is the log2 of the expression of each gene. [file peerj-08-8763-s002.png]

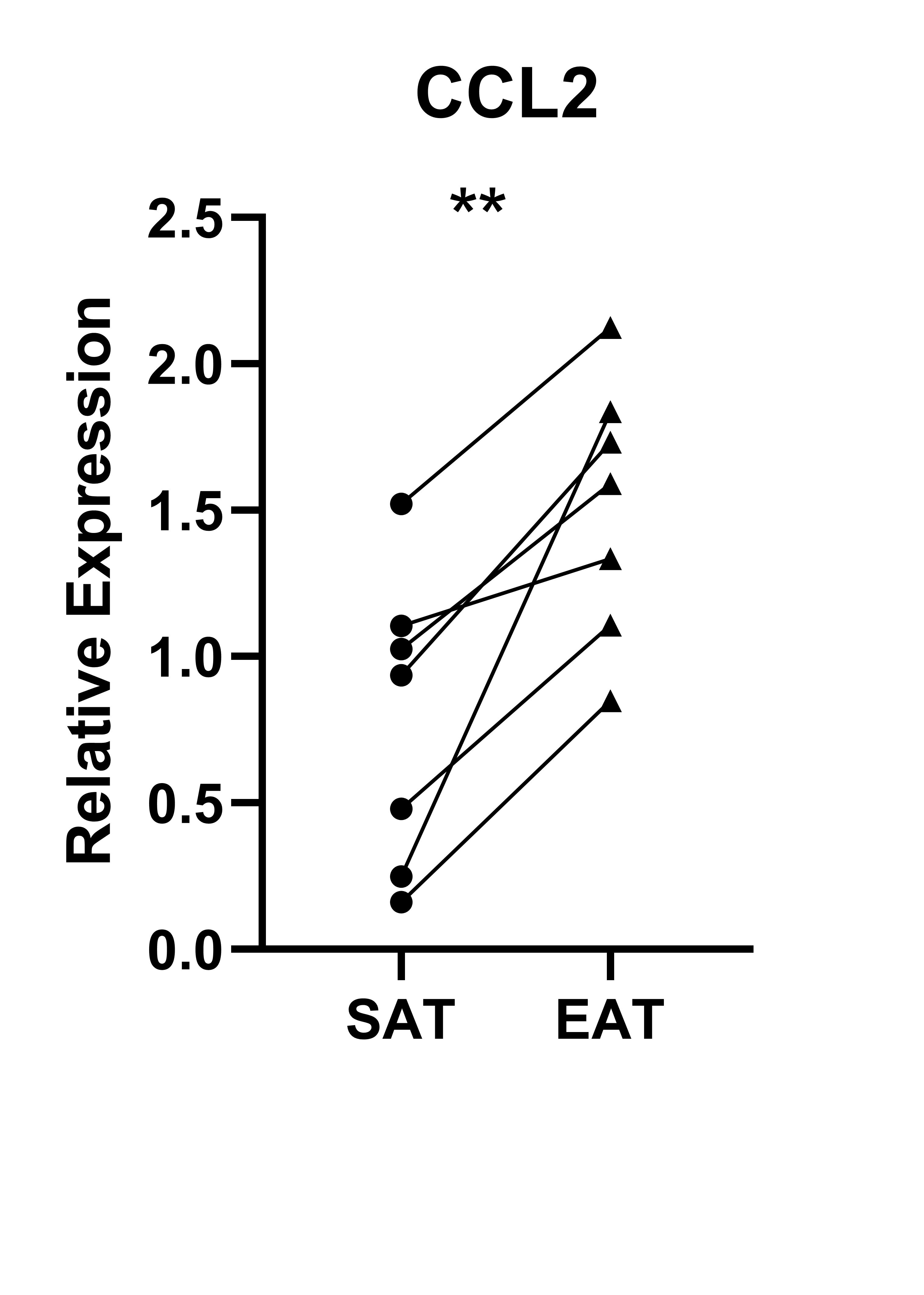

Supplement: Supplemental Information 10 [file peerj-08-8763-s010.zip › rawdata-RT-PCR/CCL2.jpg]

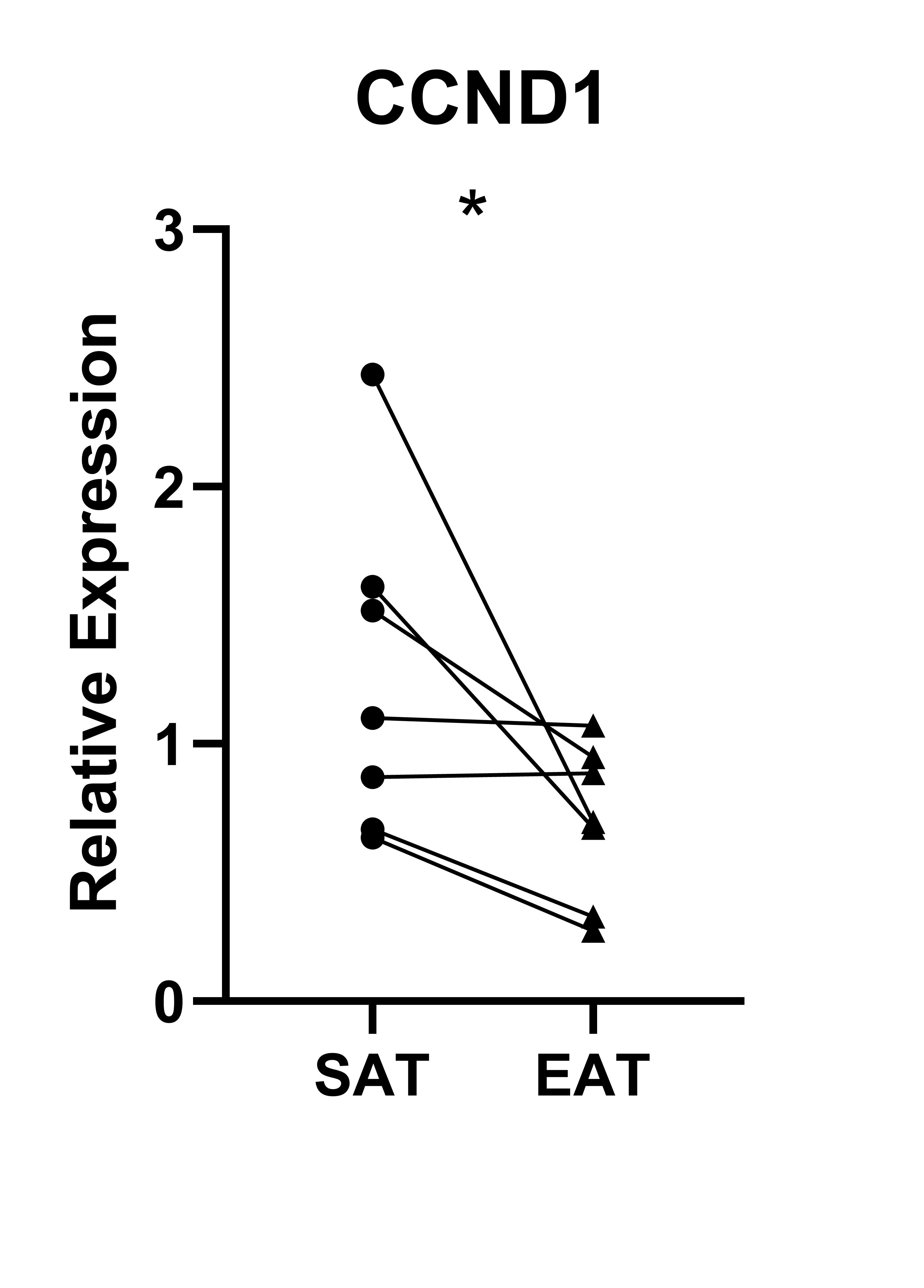

Supplement: Supplemental Information 10 [file peerj-08-8763-s010.zip › rawdata-RT-PCR/CCND1.jpg]

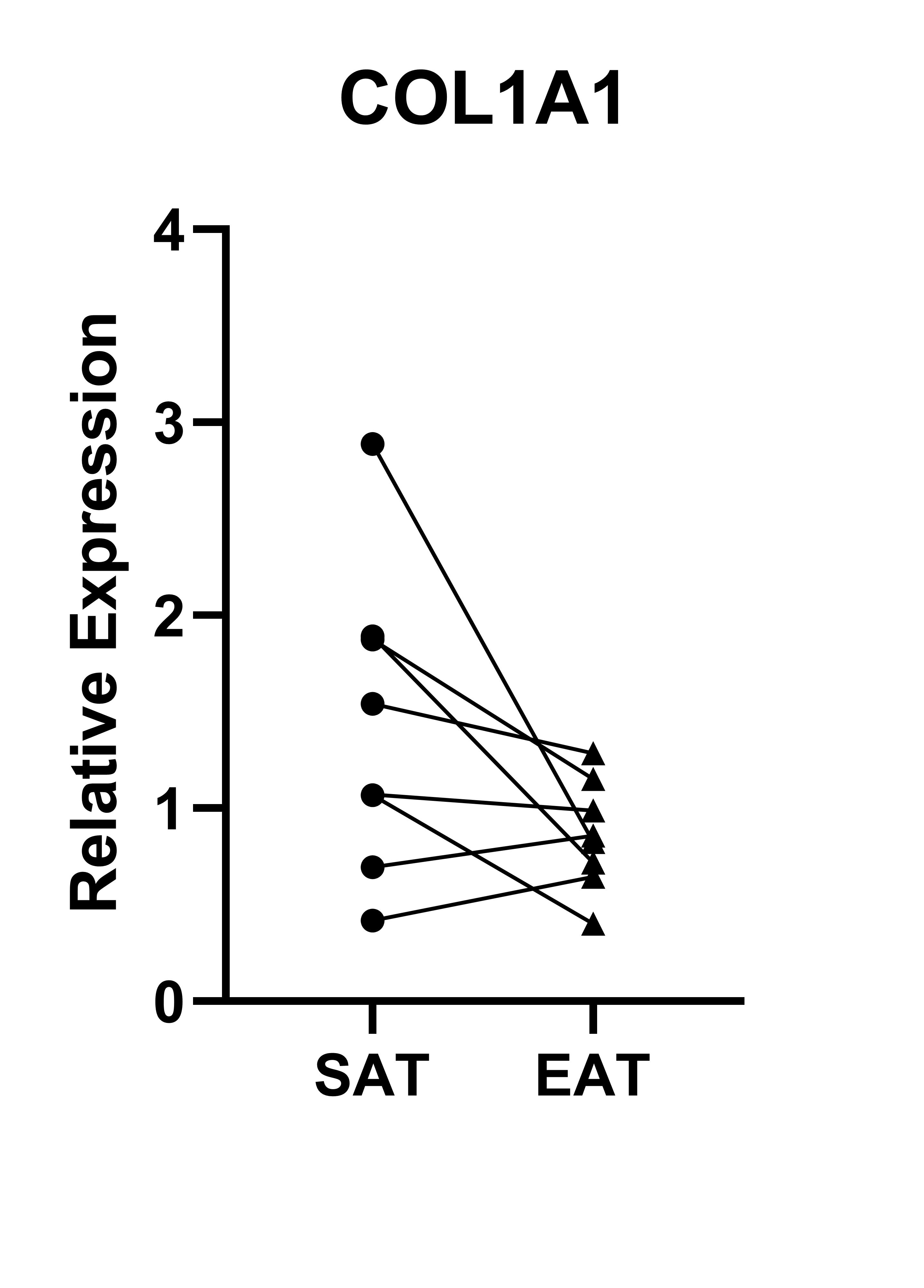

Supplement: Supplemental Information 10 [file peerj-08-8763-s010.zip › rawdata-RT-PCR/COL1A1.jpg]

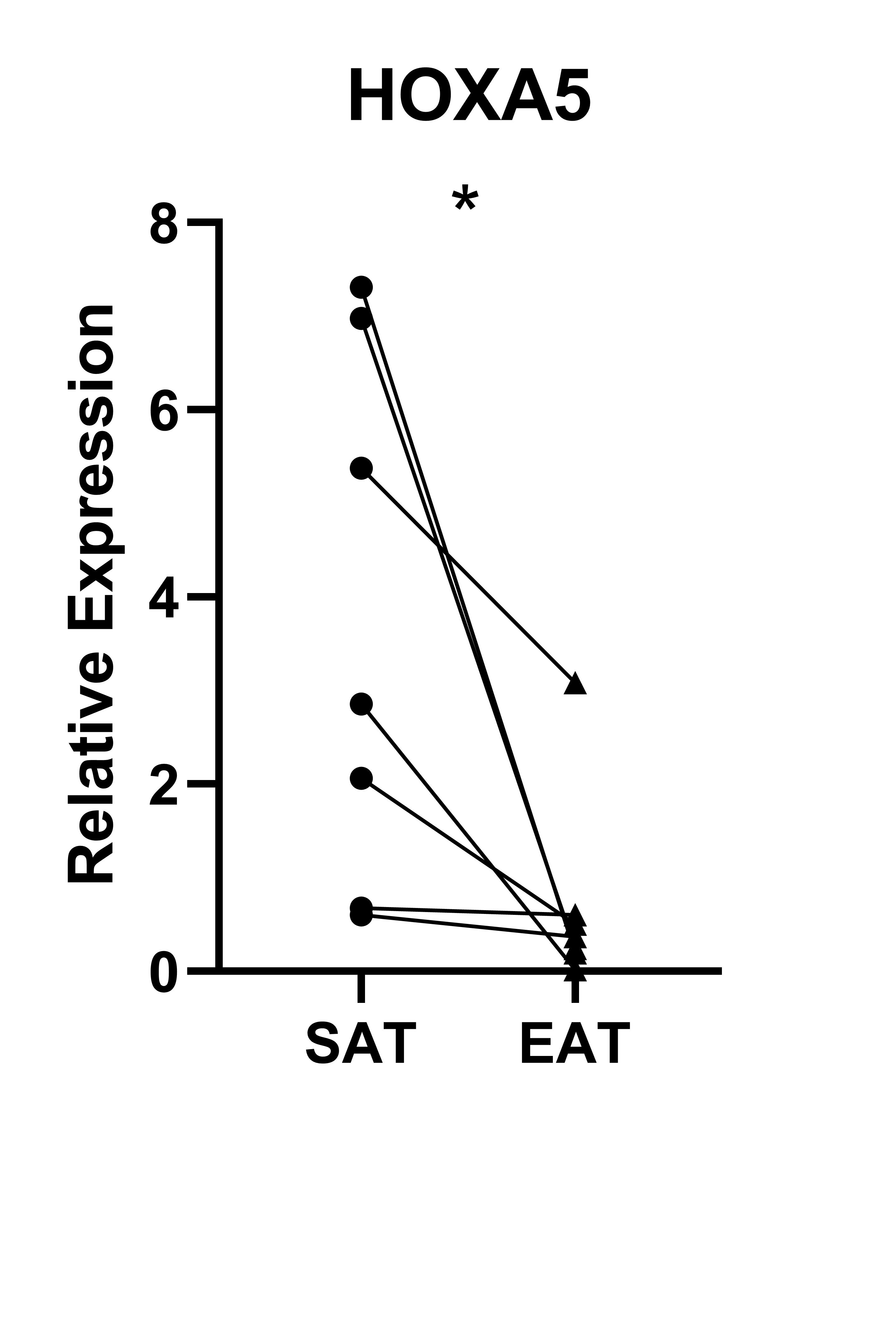

Supplement: Supplemental Information 10 [file peerj-08-8763-s010.zip › rawdata-RT-PCR/HOXA5.jpg]

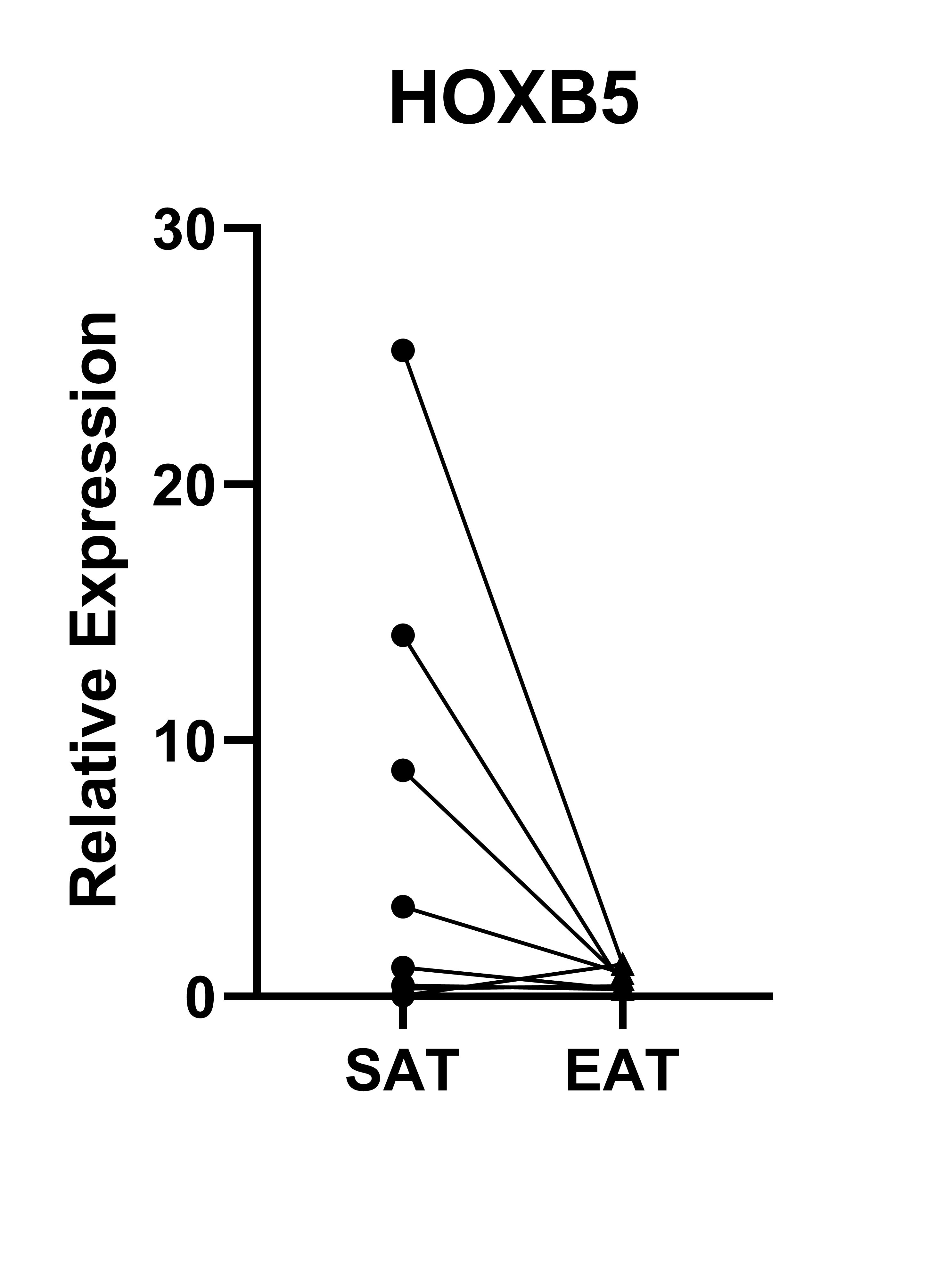

Supplement: Supplemental Information 10 [file peerj-08-8763-s010.zip › rawdata-RT-PCR/HOXB5.jpg]

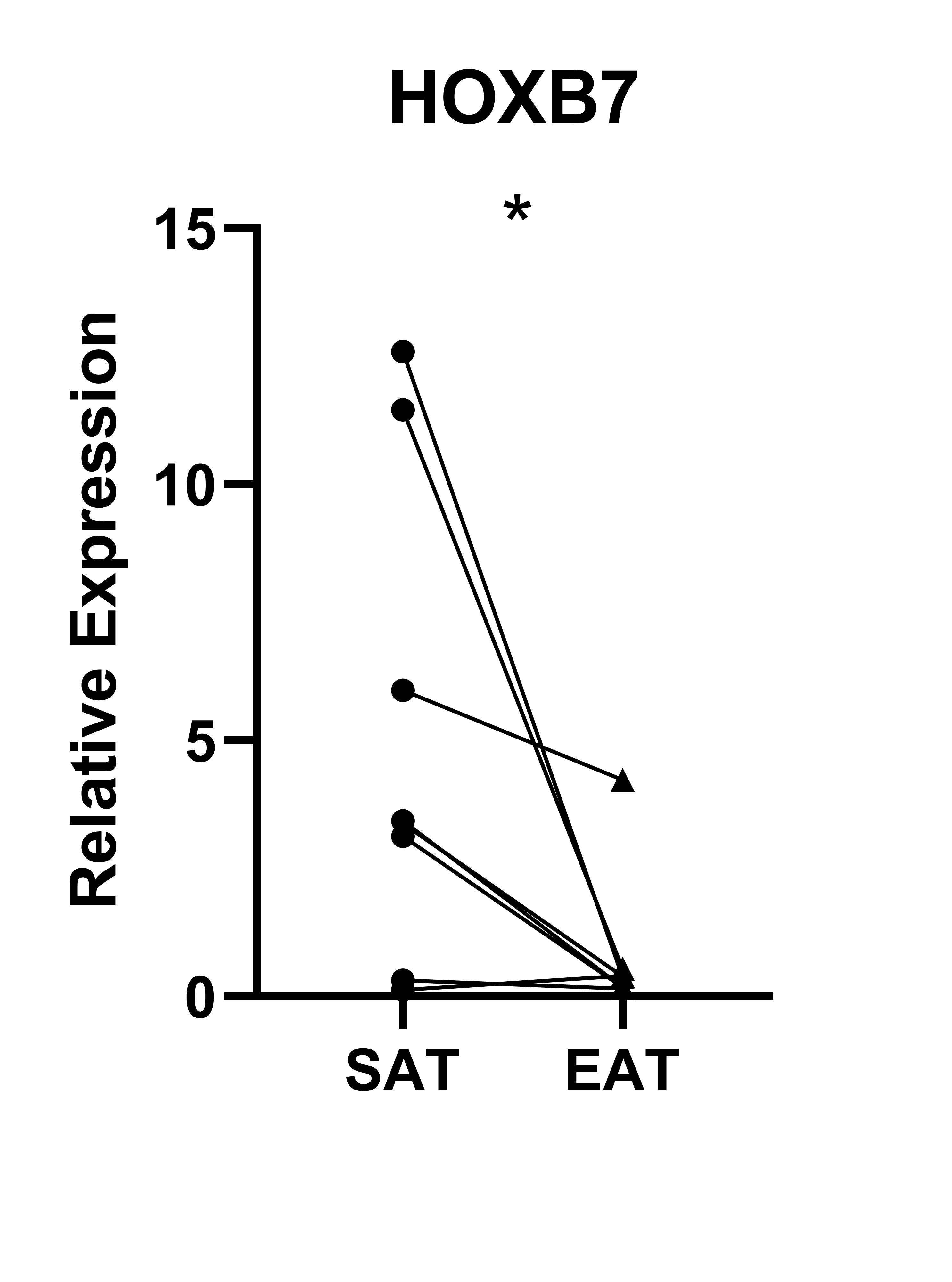

Supplement: Supplemental Information 10 [file peerj-08-8763-s010.zip › rawdata-RT-PCR/HOXB7.jpg]

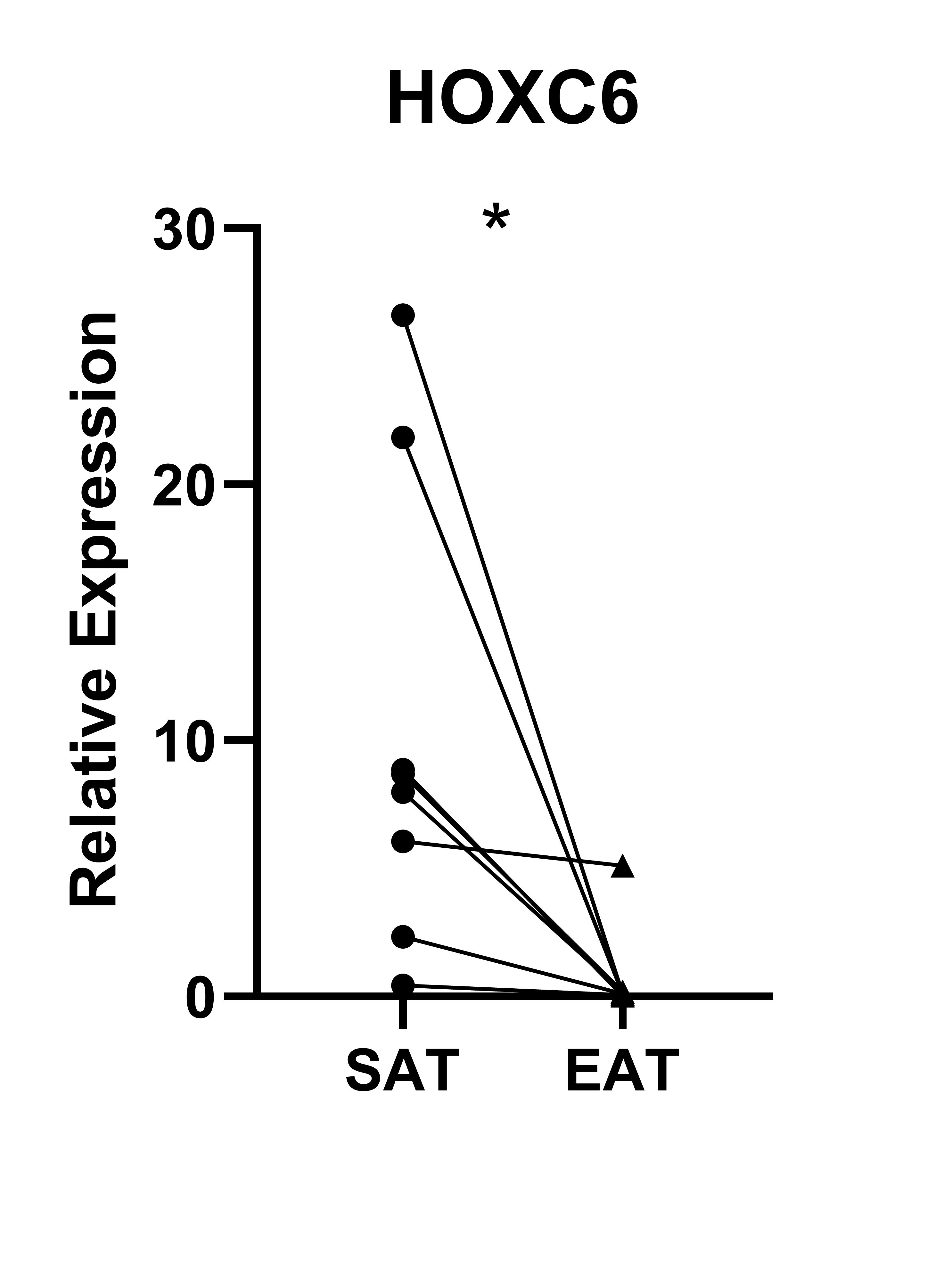

Supplement: Supplemental Information 10 [file peerj-08-8763-s010.zip › rawdata-RT-PCR/HOXC6.jpg]

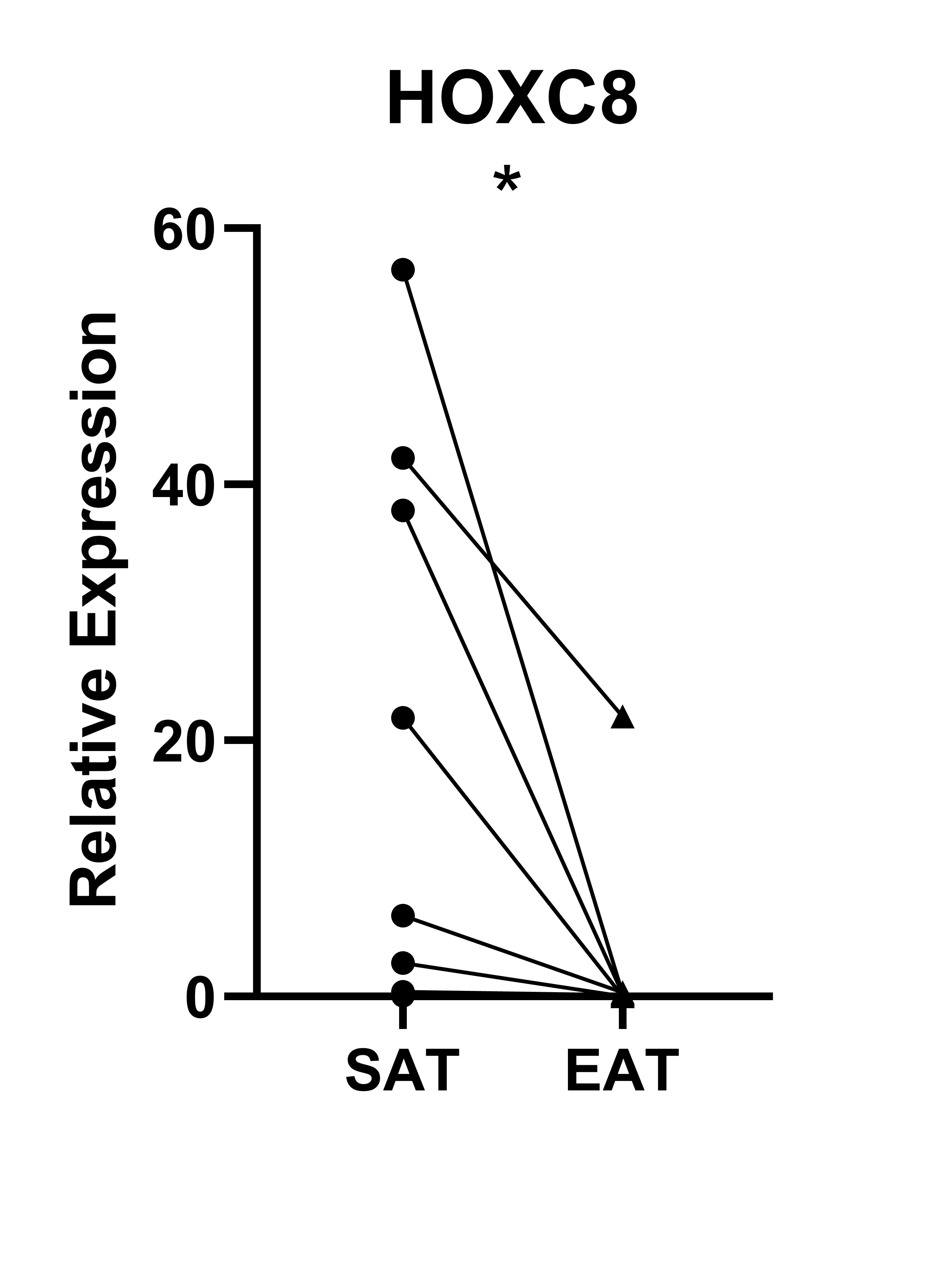

Supplement: Supplemental Information 10 [file peerj-08-8763-s010.zip › rawdata-RT-PCR/HOXC8.jpg]

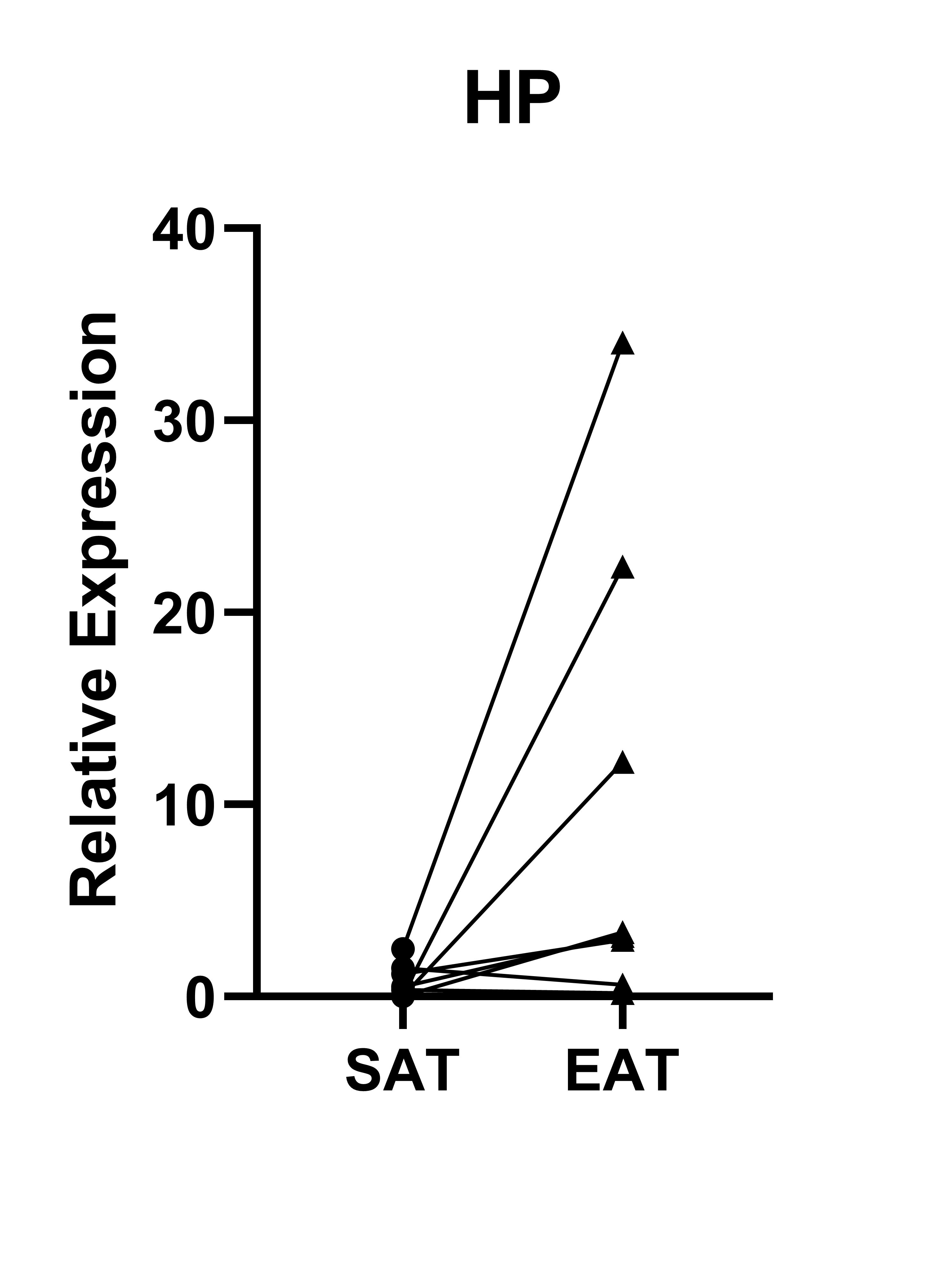

Supplement: Supplemental Information 10 [file peerj-08-8763-s010.zip › rawdata-RT-PCR/HP.jpg]

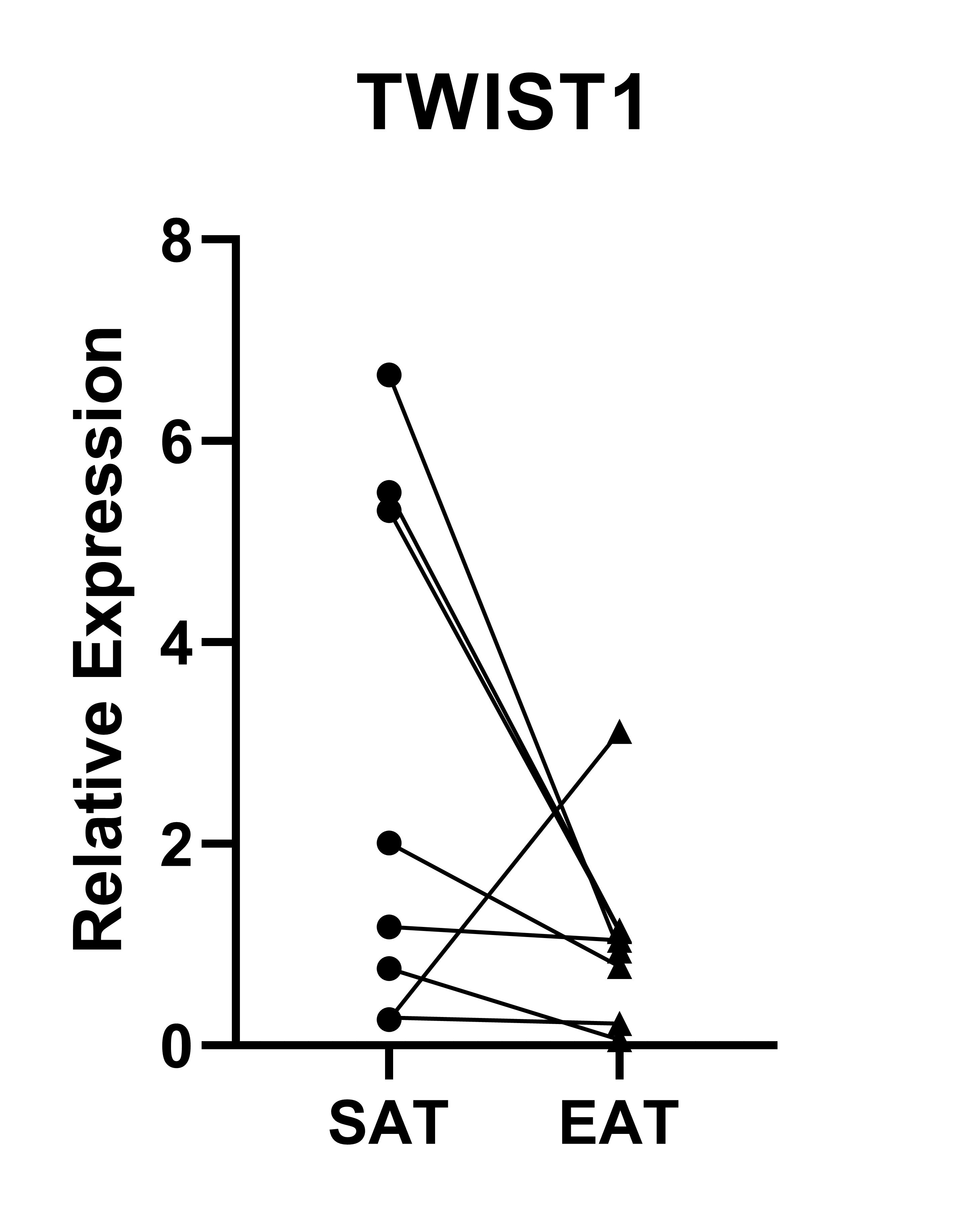

Supplement: Supplemental Information 10 [file peerj-08-8763-s010.zip › rawdata-RT-PCR/TWIST1.jpg]
