# Supplementary material for: Identification of key genes and pathways affected in epicardial adipose tissue from patients with coronary artery disease by integrated bioinformatics analysis
Source: PeerJ. 2020 Mar 25;8:e8763. doi: 10.7717/peerj.8763 (PMC7102503; doi:10.7717/peerj.8763)
Supplement: Supplemental Information 3 [file peerj-08-8763-s003.docx]

| **ID** | **Description** | **P value** | **Count** |
| --- | --- | --- | --- |
| GO:0001664  GO:0008009  GO:0042379  GO:0005501  GO:0019840  GO:0048020  GO:0016918  GO:0005126  GO:0005125  GO:0031720  GO:0140104  GO:0016004  GO:0000980  GO:0030414  GO:0004857  GO:0003705  GO:0016775  GO:0060229  GO:0005344  GO:0019841  GO:0001158  GO:0050780  GO:0035326  GO:0061134  GO:0005539  GO:0005201 | G protein-coupled receptor binding  chemokine activity  chemokine receptor binding  retinoid binding  isoprenoid binding  CCR chemokine receptor binding  retinal binding  cytokine receptor binding  cytokine activity  haptoglobin binding  molecular carrier activity  phospholipase activator activity  RNA polymerase II distal enhancer  sequence-specific DNA binding  peptidase inhibitor activity  enzyme inhibitor activity  transcription factor activity, RNA polymerase II distal enhancer sequence-specific binding  phosphotransferase activity,  nitrogenous group as acceptor  lipase activator activity  oxygen carrier activity  retinol binding  enhancer sequence-specific DNA  binding  dopamine receptor binding  enhancer binding  peptidase regulator activity  glycosaminoglycan binding  extracellular matrix structural constituent | 4.41E-07  2.76E-06  1.22E-05  1.85E-05  2.32E-05  4.24E-05  4.83E-05  0.000285284  0.000479362  0.000893511  0.000973928  0.001088857  0.001107421  0.001426724  0.001468005  0.001472661  0.001535129  0.001535129  0.001785721  0.002054394  0.002101628  0.002645325  0.003143301  0.003253732  0.003381978  0.005415207 | 10  5  5  4  4  4  3  7  6  2  3  2  4  5  7  4  2  2  2  2  4  2  4  5  5  4 |

**Supplymental Table S1：**All significant GO pathways enriched by DEGs.
