# Supplementary material for: Identification of key genes and pathways affected in epicardial adipose tissue from patients with coronary artery disease by integrated bioinformatics analysis
Source: PeerJ. 2020 Mar 25;8:e8763. doi: 10.7717/peerj.8763 (PMC7102503; doi:10.7717/peerj.8763)
Supplement: Supplemental Information 4 [file peerj-08-8763-s004.docx]

| **Terms** | **ES** | **NES** | **NOM**  **-p-val** |
| --- | --- | --- | --- |
| KEGG SPHINGOLIPID METABOLISM  KEGG NOD LIKE RECEPTOR SIGNALING PATHWAY  KEGG TYPE I DIABETES MELLITUS  KEGG LONG TERM DEPRESSION  KEGG GRAFT VERSUS HOST DISEASE  KEGG CELL ADHESION MOLECULES CAMS  KEGG ALLOGRAFT REJECTION  KEGG INTESTINAL IMMUNE NETWORK FOR IGA PRODUCTION  KEGG PRION DISEASES  KEGG LEISHMANIA INFECTION  KEGG CHEMOKINE SIGNALING PATHWAY  KEGG COMPLEMENT AND COAGULATION CASCADES  KEGG ALPHA LINOLENIC ACID METABOLISM  KEGG SYSTEMIC LUPUS ERYTHEMATOSUS  KEGG GNRH SIGNALING PATHWAY  KEGG B CELL RECEPTOR SIGNALING PATHWAY  KEGG PRIMARY IMMUNODEFICIENCY  KEGG FC EPSILON RI SIGNALING PATHWAY  KEGG HEMATOPOIETIC CELL LINEAGE  KEGG T CELL RECEPTOR SIGNALING PATHWAY | 0.55  0.72  0.71  0.52  0.75  0.57  0.74  0.72  0.71  0.63  0.47  0.65  0.57  0.55  0.42  0.53  0.72  0.46  0.56  0.47 | 2.2  2.1  2.09  2.08  2.01  2  1.97  1.86  1.85  1.82  1.75  1.73  1.73  1.71  1.67  1.65  1.64  1.63  1.62  1.59 | 0.006  0.000  0.004  0.004  0.006  0.006  0.002  0.002  0.002  0.01  0.006  0.015  0.024  0  0.01  0.014  0.025  0.018  0.041  0.028 |

**Supplymental Table S2:** Top 20 significant KEGG pathways enriched by DEGs in GSEA.
