# Supplementary material for: Identification of key genes and pathways affected in epicardial adipose tissue from patients with coronary artery disease by integrated bioinformatics analysis
Source: PeerJ. 2020 Mar 25;8:e8763. doi: 10.7717/peerj.8763 (PMC7102503; doi:10.7717/peerj.8763)
Supplement: Supplemental Information 5 [file peerj-08-8763-s005.docx]

| **Terms** | **ES** | **NES** | **NOM-p-val** |
| --- | --- | --- | --- |
| GO LYMPHOCYTE MIGRATION  GO HUMORAL IMMUNE RESPONSE MEDIATED  BY CIRCULATING IMMUNOGLOBULIN  GO LYMPHOCYTE CHEMOTAXIS  GO LYMPHOCYTE MEDIATED IMMUNITY  GO ANTIGEN BINDING  GO CCR CHEMOKINE RECEPTOR BINDING  GO ADAPTIVE IMMUNE RESPONSE  GO ADAPTIVE IMMUNE RESPONSE BASED ON SOMATIC RECOMBINATION OF IMMUNE  RECEPTORS BUILT FROM IMMUNOGLOBULIN SUPERFAMILY DOMAINS  GO PEPTIDE ANTIGEN BINDING  GO B CELL MEDIATED IMMUNITY  GO CELLULAR RESPONSE TO INTERFERON  GAMMA  GO PROTEIN ACTIVATION CASCADE  GO B CELL RECEPTOR SIGNALING PATHWAY  GO REGULATION OF GLIOGENESIS  GO DENDRITIC CELL MIGRATION  GO COMPLEMENT ACTIVATION  GO REGULATION OF ACUTE INFLAMMATORY RESPONSE  GO REGULATION OF LEUKOCYTE  PROLIFERATION  GO REGULATION OF VASCULAR ENDOTHELIAL GROWTH FACTOR PRODUCTION  GO REGULATION OF PROTEIN ACTIVATION CASCADE | 0.8  0.81  0.81  0.64  0.7  0.8  0.6  0.66  0.79  0.7  0.61  0.7  0.76  0.6  0.87  0.77  0.63  0.54  0.79  0.76 | 2.47  2.4  2.36  2.35  2.34  2.34  2.33  2.32  2.24  2.22  2.21  2.2  2.18  2.18  2.17  2.17  2.17  2.15  2.15  2.14 | 0.000  0.000  0.000  0.000  0.000  0.000  0.000  0.000  0.000  0.000  0.000  0.000  0.000  0.000  0.000  0.000  0.000  0.000  0.000  0.000 |

**Supplymental Table S3:** Top 20 significant GO pathways enriched by DEGs in GSEA.
