# Supplementary material for: Identification of key genes and pathways affected in epicardial adipose tissue from patients with coronary artery disease by integrated bioinformatics analysis
Source: PeerJ. 2020 Mar 25;8:e8763. doi: 10.7717/peerj.8763 (PMC7102503; doi:10.7717/peerj.8763)
Supplement: Supplemental Information 9 [file peerj-08-8763-s009.zip › index2.html]

Index for xtools.gsea.Gsea my\_analysis.Gsea.1557974074582

### GSEA Report for Dataset input

#### Enrichment in phenotype: **EAT (28 samples)**

- 2222 / 4322 gene sets are upregulated in phenotype **EAT**- 507 gene sets are significant at FDR < 25%- 432 gene sets are significantly enriched at nominal pvalue < 1%- 611 gene sets are significantly enriched at nominal pvalue < 5%- Snapshot of enrichment results- Detailed enrichment results in html format- Detailed enrichment results in excel format (tab delimited text)- Guide to interpret results

#### Enrichment in phenotype: **SAT (27 samples)**

- 2100 / 4322 gene sets are upregulated in phenotype **SAT**- 96 gene sets are significantly enriched at FDR < 25%- 223 gene sets are significantly enriched at nominal pvalue < 1%- 343 gene sets are significantly enriched at nominal pvalue < 5%- Snapshot of enrichment results- Detailed enrichment results in html format- Detailed enrichment results in excel format (tab delimited text)- Guide to interpret results

#### Dataset details

- The dataset has 14324 features (genes)- No probe set => gene symbol collapsing was requested, so all 14324 features were used

#### Gene set details

- Gene set size filters (min=15, max=500) resulted in filtering out 1595 / 5917 gene sets- The remaining 4322 gene sets were used in the analysis- List of gene sets used and their sizes (restricted to features in the specified dataset)

#### Gene markers for the **EAT** *versus* **SAT** comparison

- The dataset has 14324 features (genes)- # of markers for phenotype **EAT**: 6116 (42.7% ) with correlation area 45.2%- # of markers for phenotype **SAT**: 8208 (57.3% ) with correlation area 54.8%- Detailed rank ordered gene list for all features in the dataset- Heat map and gene list correlation  profile for all features in the dataset

#### Global statistics and plots

- Plot of p-values *vs.* NES- Global ES histogram

#### Other

- Parameters used for this analysis

#### Comments

- There were duplicate row identifiers in the specified dataset. One id was arbitarilly choosen. Details are below
  Generally, this is OK, but if you want to avoid this automagic, edit your dataset so that all row ids are unique
  # of row ids in original dataset: 14914
  # of row UNIQUE ids in original dataset: 14324
  # The duplicates were
  SLC25A37
  FBXO9
  ARID5A
  PPP2R3B
  ZNF277
  TARP
  TTC6
  C2
  ERCC3
  GK
  IDS
  KRT18
  MICA
  NF1
  PTEN
  TYR
  KRT17
  KRT17
  PECAM1
  ATRX
  FCGR1A
  FCGR1A
  FCGR3A
  LTA
  LTA
  RPL9
  AMY2A
  CACNA1C
  CHRNA7
  DHFR
  DHFR
  DRD5
  FKBP1A
  IGF1R
  NPR3
  P4HB
  RPL5
  RPL7
  RPL12
  RPL12
  RPL13
  RPL18A
  RPL21
  RPL21
  RPL23A
  RPL17
  RPL24
  RPL34
  REEP3
  TBC1D3B
  TBC1D3B
  TBC1D3B
  OR2W3
  RPLP0
  ANAPC11
  FCGR2B
  KCTD11
  SMAD2
  RABL2B
  GTF2IRD2B
  OR2M7
  OR4C46
  OR4D10
  OR4M2
  OR2J3
  OR2J3
  DAZ4
  OR8B3
  OR4K15
  OR4A47
  SUMO2
  SUMO2
  RPS3A
  RPS3A
  RBMY1J
  RBMY1J
  RBMY1J
  RBMY1J
  LRRC37A2
  LRRC37A2
  LRRC37A2
  CACYBP
  MOG
  PRAMEF4
  PRAMEF4
  B3GNTL1
  RPS7
  PCGF6
  RPSA
  ROPN1B
  ANKRD20A3
  ANKRD20A3
  ANKRD20A3
  LSP1
  DDR2
  FOLH1
  BTBD11
  HFM1
  DKK3
  FRMPD2
  GOLGA8B
  RGPD1
  RGPD1
  RGPD1
  RGPD1
  RGPD1
  HIST2H2BF
  HIST2H2BF
  SH3KBP1
  CBWD5
  CBWD5
  CBWD5
  RPS25
  RPS26
  RPS26
  RPS26
  RPS26
  RPS27
  RPS27
  CPLX3
  BOLA2
  GLUL
  TOM1L2
  RPL14
  RPL41
  RPL41
  SDHC
  DYNLL1
  NBPF9
  NBPF9
  NBPF9
  MARVELD2
  YIF1B
  NBPF10
  LOC650293
  LOC650293
  ANXA8
  ANXA8
  BTNL8
  ZXDC
  GAGE1
  GAGE1
  PTPN20
  PTPN20
  TPM3
  UGT2B11
  ZNF626
  PGA3
  PGA3
  TMEM183B
  CASZ1
  RASA4
  RASA4
  ZBTB38
  SLC45A4
  DYNC2H1
  TSPAN11
  KIR2DL4
  PXN
  SPAG11A
  FAM86B1
  FAM86B1
  MORC4
  MORC4
  SFTPA1
  ACR
  GPR89A
  POLR2J3
  HLA-F
  HLA-F
  RAPGEF3
  SFTPA2
  ZBTB34
  PTMA
  PTMA
  PTMA
  PTMA
  ALG13
  PACS2
  DGKZ
  CCHCR1
  NBEAL1
  SET
  CCR2
  CFLAR
  AURKAIP1
  VPS53
  EEF1D
  ACAA1
  FAM58A
  CDV3
  ZDHHC3
  RPS27A
  RPS27A
  AGER
  BTF3L4
  SYT2
  LRRFIP1
  LRRFIP1
  LRRFIP1
  RGSL1
  DAXX
  WEE1
  DET1
  ANKRD30B
  RASGRF1
  SORBS2
  BIVM
  NRG1
  ZNF138
  ARMC10
  C6orf136
  CSF2RA
  COL11A2
  ANKRD36
  PTCH2
  SRGAP2
  IQSEC3
  IQSEC3
  ABCC6
  ARHGDIG
  SCN8A
  PIGT
  VDAC2
  WNK1
  DNAJC19
  CLSPN
  UNKL
  DHRS4L2
  DENND1B
  GABPA
  HLA-DQB2
  SERINC2
  AK2
  RPL17
  CNTNAP3B
  CNTNAP3B
  CNTNAP3B
  DDR1
  ATP6V1G2
  SRP19
  ZNF587
  RAB43
  USMG5
  LOC554223
  HLA-DPA1
  GFOD1
  HLA-A
  TRIM26
  HLA-C
  HLA-C
  FYB
  HIP1
  GNG2
  HDLBP
  HLA-DQB1
  HLA-DQB1
  HLA-DRB1
  LCN1
  RPL15
  ANKRD11
  MSTO1
  RPL10
  RNF165
  DDX11
  DDX11
  CCDC74A
  PDPK1
  CDSN
  FBXW10
  RPS3A
  CTCFL
  RXRB
  RXRB
  WTAP
  HYDIN
  HYDIN
  HYDIN
  KIF27
  KIF27
  MEGF8
  RAB13
  AP1S2
  GPSM3
  SDHD
  IGSF9B
  AOC3
  ALDH6A1
  RAD17
  ZNRD1
  ABCB8
  ABCB8
  ABCB8
  CSNK2B
  MAGEA2
  CDRT1
  TRA2A
  SERHL2
  VKORC1L1
  TPT1
  CNOT6L
  CBWD5
  CBWD5
  CBWD5
  D2HGDH
  DKC1
  SDAD1
  EFCAB2
  KIR2DS2
  KIR2DS2
  KIR2DS2
  TUBB
  TUBB
  DUX4
  DUX4
  DUX4
  DUX4
  DUX4
  SDHA
  EEF1A1
  EIF4B
  EIF4B
  GPR32
  CKS1B
  EIF4A2
  FGF7
  FTH1
  GAB1
  GNA11
  H3F3A
  H3F3A
  H3F3A
  HLA-DMB
  HLA-DOB
  HLA-DPB1
  HLA-DPB1
  HLA-DQA1
  HLA-G
  HMGB1
  HSPA6
  KCNG1
  MAFG
  MAFG
  MOG
  PBX2
  PLCG2
  POU5F1
  PRSS2
  PSMC6
  RBMS1
  RING1
  RPS2
  ST8SIA1
  SNRPE
  SNRPG
  SRP9
  SSRP1
  TAPBP
  TAPBP
  TERF1
  TPSAB1
  TSPYL1
  UBE2L3
  HIST1H2AI
  HIST1H4K
  DOC2B
  DHX16
  ADAM21
  NRP2
  GBP2
  STK19
  GCSH
  GCSH
  HERC2
  HERC2
  HERC2
  CD8B
  HMGN1
  KIF5A
  PRB1
  GNG5
  GNL1
  GNL1
  GRK5
  RAPGEF1
  PDIA3
  HSPA1A
  HSPA1B
  RBPJ
  MAGEA3
  TOM1L1
  HLA-B
  HLA-E
  HMGN2
  HMGN2
  KRT6A
  RHEB
  MMP16
  MT2A
  MT2A
  UQCRFS1
  VARS
  ANP32A
  NPEPPS
  RAN
  TIMM23
  RBM14
  SEMA4D
  CDRT1
  ARID3B
  AVIL
  MTHFD2
  TCEA1
  YWHAE
  TRIM10
  IFNA13
  RNF5
  SLC5A3
  TRIM31
  TRIM31
  TCF19
  ZNF90
  LYPLA2
  LYPLA2
  ACOT7
  ELL2
  ARL2BP
  FBXO25
  RHOQ
  FBXO9
  OR11A1
  DEXI
  ARHGEF10
  PLEKHM1
  AAK1
  FRYL
  TBC1D2B
  SMG1
  SMG1
  SMG1
  SMG1
  SMG1
  BOP1
  PRUNE2
  MICAL3
  SMCHD1
  PSD3
  AHCTF1
  GPATCH4
  C1QTNF5
  PELO
  KLF13
  FAHD2A
  RPL26L1
  IER3IP1
  SELT
  GLRX5
  ZCCHC2
  IMPAD1
  MRPL20
  RNF31
  FAM90A1
  PLCXD1
  RRN3
  RRN3
  PXMP2
  CLN8
  FAM35A
  TNXB
  TNXB
  HLA-DRA
  PCNP
  CD177
  CLK4
  KIAA1328
  MST1
  MTMR3
  TMSB4X
  TMSB4X
  TMSB4X
  PPIA
  LY6G5B
  GABBR1
  PPP1R11
  PGM5
  PGM5
  EIF4H
  TOR1AIP2
  SUDS3
  CSH2
  ANAPC1
  DGCR14
  NUCKS1
  HAPLN4
  UGT2A3
  FBXO17
  C6orf25
  C6orf25
  LY6G5C
  SAA2
  ITFG1
  OR2J2
  ISCA1
  SCRT1
  ING5
  THOC3
  PNMA6A
  ZNF479
  CGB1
  ZFHX2
  TRERF1
  RFT1
  ADAMTSL1
  RBM33
  HIST1H2BK
  TREX2
  ZNF618
  KIAA2013
  CYHR1
  CDC26
  TCP10L
  DNHD1
  DNHD1
  DNHD1
  LSM14B
  LSM14B
  LSM12
  ZNF75A
  KIR3DL3
  KIR3DL3
  DNAH17
  DNAH17
  FSIP2
  ARHGAP27
  TUBB8
  LCN8
  ZNF616
  ZNF678
  SLCO4C1
  KRTAP10-4
  PHLDB3
  STMN1
  ERICH1
  DNAH10
  RPL23AP7
  RPL23AP7
  TTTY14
  OR7E37P
  OR7E37P
  OR7E37P
  OR7E37P
  OR7E37P
  OR7E37P
  OR7E37P
  MEIS3P1
  WDR74
  MALAT1
  MGC72080
  PMCHL1
  FLJ25758
  FAM99A
  KIAA0125
  DBF4B
  LOC441081
  LOC441081
  FER1L4
  ESR1
  DUX4
  CCNL2
  UBQLN4
  POLR2J
  VAC14
  PPM1K
  CD99
  PDPK1
  EPHA10
  PGM5
  GPC2
  TRIM74
  VTI1A
  DNAJC9
  PRAMEF4
  HLA-A
  ALDH1A3
  AQP7
  AQP7
  SLC6A8
  RPL23AP7
  LOC401357
  CARD8- Timestamp used as random seed: 1557974095973

---

Report: my\_analysis.Gsea.1557974074582.rpt   by user: DELL

xtools.gsea.Gsea [Thu, May 16, '19 10 AM 34]

Website: www.gsea-msigdb.org/gsea
Questions & Suggestions: Contact page
